# Electrical Control of Intersubband Transitions in Few-Layer WSe_2_ Multivalley Quantum Wells Probed by Electronic Raman Scattering

**DOI:** 10.1021/acsnano.5c08378

**Published:** 2026-01-24

**Authors:** Philipp Wutz, Yinong Zhang, Felix Hofmann, Paulo E. Faria Junior, Yao Lu, Philip Soul, Yu-Han Bao, Kenji Watanabe, Takashi Taniguchi, Jaroslav Fabian, Sebastian Bange, John M. Lupton, Kai-Qiang Lin

**Affiliations:** † Institute for Experimental and Applied Physics, 9147 University of Regensburg, Regensburg 93053, Germany; ‡ Department of Physics, 7284 University of Washington, Seattle, Washington 98195-1560, United States; § Institute for Theoretical Physics, 9147 University of Regensburg, Regensburg 93053, Germany; ∥ Department of Physics, University of Central Florida, Orlando, Florida 32816, United States; ⊥ Department of Electrical and Computer Engineering, University of Central Florida, Orlando, Florida 32816, United States; # State Key Laboratory of Physical Chemistry of Solid Surfaces, College of Chemistry and Chemical Engineering, 12466 Xiamen University, Xiamen 361005, China; ∇ Research Center for Electronic and Optical Materials, 52747 National Institute for Materials Science, 1-1 Namiki, Tsukuba 305-0044, Japan; ○ Research Center for Materials Nanoarchitectonics, National Institute for Materials Science, 1-1 Namiki, Tsukuba 305-0044, Japan

**Keywords:** 2D semiconductors, quantum wells, intersubband transitions, IR spectroscopy, electronic Raman scattering, Stark spectroscopy

## Abstract

Semiconducting quantum wells have enabled revolutionary applications in diode lasers, IR photodetectors, and optical modulators. Recently, van der Waals (vdW) quantum wells have emerged as a promising frontier, offering inherently atomically sharp interfaces and facile integration into device structures without the constraints of lattice matching. Tunability of intersubband transitions is essential for applications of quantum wells but remains unexplored in vdW structures. Here, we report valley-selective, electric-field-activated electronic Raman scattering from intersubband transitions in natural WSe_2_ multilayers and demonstrate electrical tunability by over 100 meV. We validate the generality of such tunability in 3 to 7 layers of WSe_2_ and quantify the effective dipole moments and polarizabilities that determine the quantum-confined Stark effect. These intersubband transitions are also found in artificially stacked multilayers, where they can be manipulated by twist angle. Our work lays foundations for exploiting vdW quantum wells in next-generation optoelectronic applications, including tunable photodiodes and atomically compact IR spectrometers.

## Graphical abstract

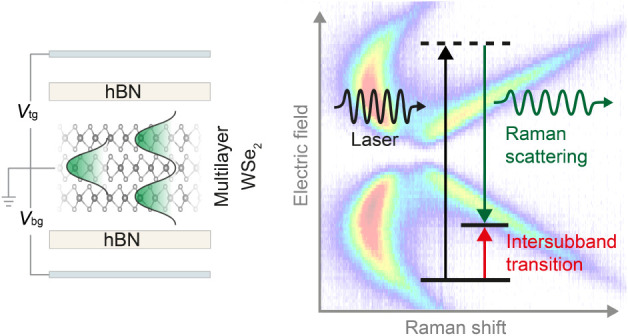

Semiconductor quantum wells have given rise to some of the most successful applications of quantum confinement and have found widespread commercial applications in optoelectronics, ranging from semiconductor lasers to IR photodetectors.^1^ The advent of van der Waals (vdW) materials has heralded a new era for quantum materials, overcoming the constraints of rigorous lattice-matching conditions in epitaxial systems.^2,3^ Few-layer vdW semiconductors have recently emerged as natural quantum-well structures, owing to their intrinsic out-of-plane confinement.^4,5^ These vdW quantum wells offer distinct advantages over their epitaxially grown counterparts, including the potential for seamless integration with a diverse range of materials and devices while at the same time providing atomically sharp interfaces. However, the field of vdW quantum wells has seen only a handful of experimental studies so far. For example, subband states in direct-bandgap few-layer InSe have been probed by photoluminescence excitation spectroscopy,^4^ resonant tunneling,^4,6^ and magnetotransport.^7^ Mid-IR excitation has recently been integrated with scanning near-field optical microscopy (SNOM) to achieve the spatial resolution necessary for probing intersubband transitions in indirect-bandgap multilayer WSe_2_ flakes.^5^ At the heart of quantum-well functionality lies the tunability of subband states and the transitions between these induced by an applied electric field oriented along the confinement direction.^8^ While this crucial feature has recently been studied theoretically in few-layer InSe,^7^ experimental demonstration remains elusive. Both THz absorption spectroscopy and near-field enhanced techniques face challenges in the presence of highly conductive top gates, which are necessary for generating out-of-plane electric fields. Electronic Raman spectroscopy, on the other hand, has proven remarkably successful in probing intersubband transitions in conventional quantum wells,^9,10^ and more recently in exploring moiré mini-bands in twisted bilayer WSe_2_.^11^ Although not yet demonstrated for intersubband transitions in vdW quantum wells, this optical technique in the visible spectral range is barely affected by the presence of semitransparent conductive gates, offering a promising avenue for further exploration.

Here, we demonstrate how electronic Raman scattering can be used to probe intersubband transitions in vdW quantum wells. Inelastic light scattering from intersubband transitions in few-layer WSe_2_ arises exclusively in the presence of an out-of-plane electric field. This phenomenon enables the concurrent study of intersubband transitions originating from multiple Brillouin-zone valleys with high-energy resolution under controlled electrostatic doping. We demonstrate the electrical tunability of intersubband transitions for resident carriers at different valleys in 3-layer WSe_2_, corroborating the results by density functional theory (DFT) calculations. We further illustrate the robustness of intersubband transitions in twisted double-bilayer WSe_2_, comparing to natural four-layer WSe_2_ to illustrate the impact of the twist angle. Finally, we establish the generality of our findings by examining natural WSe_2_ of up to 7 layers, unveiling the layer-number dependence of the energies of the intersubband transitions as well as their effective dipole moments and polarizabilities. Using first-principles calculations based on DFT and appropriate symmetry analysis, we fully elucidate the microscopic behavior of the subbands and intersubband transitions as a function of out-of-plane electric field and number of layers.

## Results

Figure 1a illustrates the dual-gate device structure of natural trilayer 2H-stacked WSe_2_, with an optical micrograph of the device shown in Figure 1. The gate configuration allows for independent tuning of the out-of-plane electric field *F*_z_ and the carrier density *n* in the semiconductor via top and bottom gate voltages.^12,13^ The band structure of three-layer WSe_2_ calculated by DFT is shown in Figure 1b to highlight conceivable intersubband transitions for resident electrons in the conduction bands (CB) at the Q valley, CB­(Q), resident holes in the valence band (VB) at the Γ valley, VB­(Γ), and resident holes in the K valley, VB­(K). We excite the device with a 488 nm CW laser and measure the scattered light as a function of *F*_z_ for two electrostatically defined doping densities in Figure 1d (hole doping, |*n*| = 4.5 × 10^12^ cm^–2^) and Figure 1e (electron doping, |*n*| = 4 × 10^12^ cm^–2^). A complete dependence on the doping density is shown in Supplementary Figure 1.

**Figure 1:**
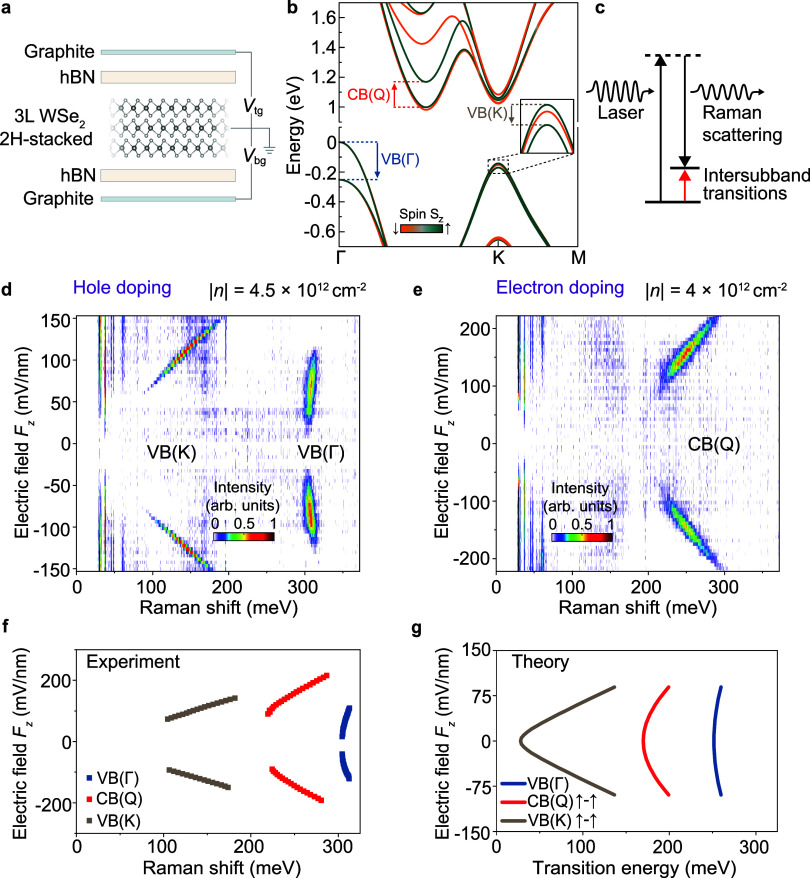
Electrically tunable intersubband transitions in three-layer WSe_2_. (a) Schematic illustration of a dual-gate 2H-stacked WSe_2_ device. Top and bottom gates (tg, bg) of few-layer graphene control the carrier density and the out-of-plane electric field via *V*_tg_ and *V*_bg_ while the WSe_2_ layer is grounded. (b) Spin-resolved band structure of the WSe_2_ trilayer at zero field, with intersubband transitions at the Γ, Q, and K valleys highlighted by arrows. (c) Simplified energy-level diagram of electronic Raman scattering. (d, e), Stokes Raman scattering as a function of out-of-plane electric field for hole doping (d) and electron doping (e) under 488 nm excitation, showing intersubband transitions at the valence band (VB) K and Γ valleys and at the conduction band (CB) Q valley as illustrated in panel b. All spectra are shown after subtraction of the zero-field spectrum for a sample temperature of 1.8 K. (f) Spectral peak positions extracted from panels d and e as a function of electric field. (g) Lowest spin-conserved intersubband transition calculated by DFT at VB­(K), VB­(Γ), and CB­(Q) as a function of the out-of-plane electric field.

In quantum wells, the first and second subbands correspond to wave functions of opposite parity with respect to the out-of-plane coordinate, and as such, transitions between them cannot be observed in inelastic Raman scattering unless that parity is broken by an applied electric field. To highlight the field-induced change in Raman scattering, Figure 1d–e and all subsequent plots show difference spectra resulting from the subtraction of the scattering spectrum at *F*_z_ = 0. The original, uncorrected spectra are shown in Supplementary Figure 2. This procedure suppresses field-independent signals such as Raman scattering from phonon modes associated with the WSe_2_, hBN, and graphite electrodes.^14,15^ As shown in Figure 1d–e, two narrow-linewidth peaks emerge in the hole-doping regime, whereas one peak appears under electron doping, matching well with the expected transitions from holes resident in either the K or Γ valleys,^16^ and electrons resident in the Q valley. None of these peaks is prominent at electric fields close to zero, and all of them brighten and blue-shift with increasing |*F*_z_|.

The *F*_z_-dependence in Figure 1d–e resembles the Stark effect on interlayer excitons,^12,13,17,18^ showing an energy shift exceeding 0.1 eV over a field variation of less than 0.1 V/nm. Such a shift is surprisingly large for a Raman scattering process.^19–21^ To unequivocally distinguish scattered radiation from excitonic emission,^22^ we perform the measurements at different excitation wavelengths. As shown in the Supporting Information in Figure 3, the emission peaks shift with excitation energy, while the Raman shifts remain the same. We summarize the variation of the scattering peak positions with the field in Figure 1f. To elucidate the field dependencies and identify the corresponding transitions, we incorporate external electric fields in our DFT calculations^13^ and evaluate intersubband transition energies for electrons in the Q valley and holes in either the K or the Γ valleys of the VB. Since Raman scattering from intersubband transitions is found to be copolarized in the helicity-resolved measurement (Supplementary Figure 4), we only consider the spin-conserved intersubband transitions^9^ in the calculations. As illustrated in Figure 1g, our DFT calculations fully reproduce the experimental results in terms of both absolute transition energies and relative electric-field-induced shifts, allowing an assignment of the corresponding transitions and validating the underlying inherent electrical tunability of vdW quantum wells. The magnitude of field-induced shifts in transition energy follows a clear hierarchy in terms of the nature of the resident carrier, VB­(Γ) > CB­(Q) > VB­(K), reflecting the degree of wave function delocalization in the respective valleys.^23–25^

Beyond the case of three-layer WSe_2_, we expect the sample response to be distinctly different in systems with an even number of layers. In these, spatial inversion symmetry is restored and broken only upon introduction of the out-of-plane electric field. Figure 2a–b shows the zero-field-corrected Raman spectra of natural 2H-stacked four-layer WSe_2_ as a function of the out-of-plane electric field *F*_z_. Uncorrected spectra are shown in Supplementary Figure 5. A complete doping dependence and excitation-energy dependence are shown in Supplementary Figures 6–7. As the number of layers increases, the semiconductor acquires a more indirect character: both energy differences |VB­(K)–VB­(Γ)| and |CB­(K)–CB­(Q)| increase^26^ (cf. Figures 1b, 2c, and Supplementary Figure 19). Resident electrons and holes should preferentially reside at a single valley (Q for electrons and Γ for holes), and one would not expect new transitions to emerge as the number of layers increases. In stark contrast to the results for 3L WSe_2_, however, two peaks emerge in the electron-doping regime, whereas only a single peak shows up under hole-doping. With increasing electric field *F*_z_, the lower-energy branch in the electron-doping regime exhibits nonmonotonic behavior—initially red-shifting and then blue-shifting again—while the higher-energy branch displays a monotonic blue shift.

**Figure 2:**
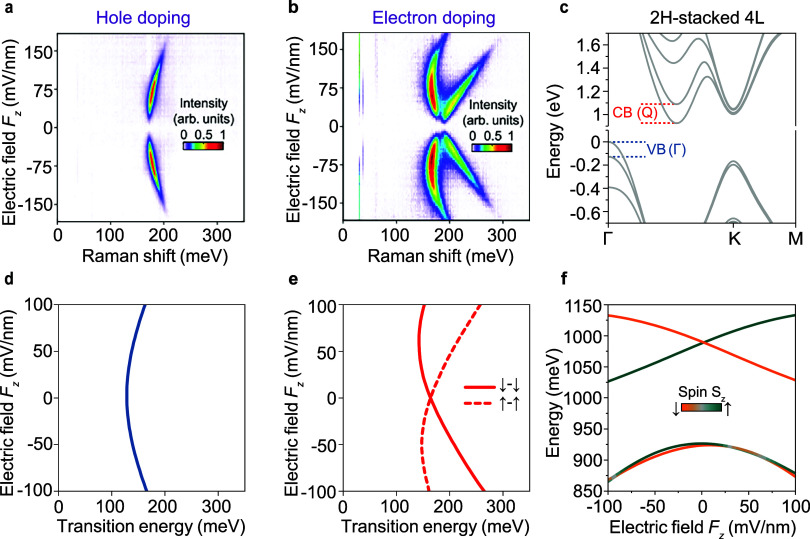
Nonmonotonic electric-field dependence of intersubband transitions in natural four-layer WSe_2_. (a, b) Stokes Raman spectra as a function of out-of-plane electric field for (a) hole doping and (b) electron doping, both at |*n*| = 5 × 10^12^ cm^–2^ under 488 nm excitation at 1.8 K. All spectra are shown with the response at zero field subtracted. (c), DFT band structure of natural 2H-stacked four-layer WSe_2_, with intersubband transitions at the Γ and Q valleys illustrated. (d, e) DFT-calculated first intersubband transition at the (d) Γ and (e) Q valleys as a function of the out-of-plane electric field. The arrows indicate the spin orientation in the layers. In the Q valleys, the spin degeneracy is lifted under the electric field. (f) Shift of the first two conduction bands at the Q valley as a function of the out-of-plane electric field. The color illustrates the spin quantum number *S*_z_.

To examine this surprising nonmonotonic behavior further, we consider the doping dependence of the field-tunable Raman shift for the natural four-layer system in Figure 3a, b. The carrier density changes neither the peak position nor the electric-field dependence of intersubband transitions, indicating that the Raman mechanism is intrinsic to the band structure and not dependent on carrier concentration. Furthermore, we observe Raman features up to a temperature of 140 K (see Supplementary Figure 8). To unveil the microscopic mechanism of this unexpected phenomenon, we performed DFT calculations of the *F*_z_-dependent band structure of 2H-stacked four-layer WSe_2_. Figure 2d–e shows the spin-conserved intersubband transition energies as a function of *F*_z_ for resident holes in the Γ valley and electrons in the Q valley, exhibiting surprisingly good agreement with the experiments. Our calculations in Figure 2e reveal that the two distinct peaks with nonmonotonic *F*_z_-dependence in Figure 2b actually follow two interpenetrating parabolas with offset minima, corresponding to the two nondegenerate spin-conserved intersubband transitions. To elucidate the origin of these parabolas, we plot the change in the energy of conduction subbands at the Q valley as a function of *F*_z_ in Figure 2f. While the first subband shows a parabolic dependence, the second subband exhibits a strong linear dependence on the electric field, giving rise to the experimentally observed nonmonotonic behavior. This functionality contrasts with the theoretical results for trilayer structures (see Supplementary Figure 9), where only a parabolic dependence is observed—a distinction that can be explained through symmetry analysis. Odd-number layered structures possess a horizontal mirror plane, resulting in a symmetric electric-field dependence of subband energies that can be characterized by a polynomial expansion with only even-order contributions. In contrast, even-numbered stacks lack a horizontal mirror plane, necessitating odd-order contributions (for detailed perturbation theory and symmetry analysis, see Supplemental Note 4.3).

**Figure 3:**
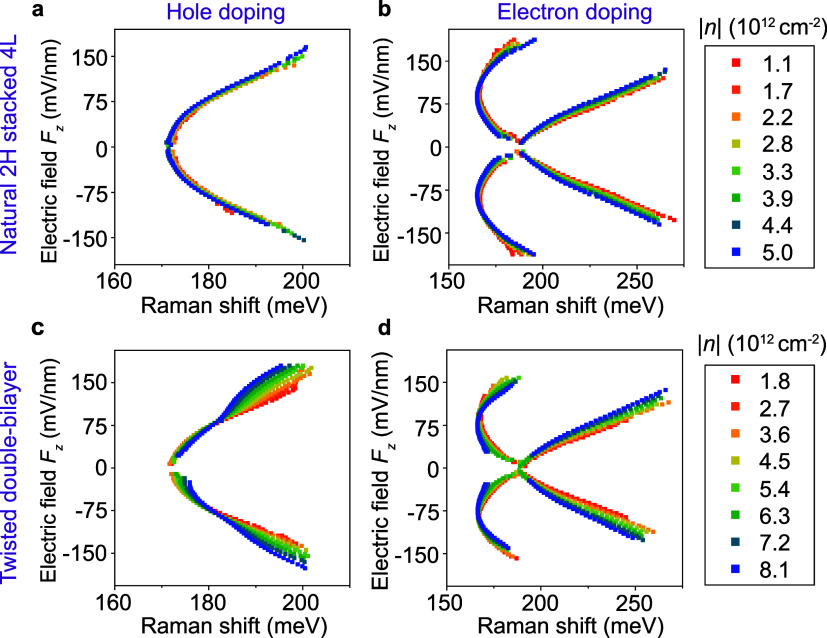
Comparison between natural four-layer WSe_2_ (a, b) and twisted double-bilayer WSe_2_ (c, d) in the doping dependence of intersubband transition energies determined by Stokes Raman scattering.

In addition to natural four-layer WSe_2_, we also studied a four-layer sample consisting of two stacked natural 2H-bilayers with the intent of testing the impact of artificial stacking as well as the influence of small deviations in twist angle on the intersubband transitions.^27^ Figure 3c, d shows the *F*_z_-dependence of the intersubband transitions in twisted double-bilayer WSe_2_ (twist angle close to 60°, i.e., close to 2H-stacking, see details in Supplementary Figure 26) at different doping densities. The results in the low-doping regime match very well with those of the natural four-layer sample in Figure 3a, b, demonstrating the robustness of the intersubband transitions against artificial stacking. Surprisingly, however, at intermediate to strong doping, an influence of doping is seen in the *F*_z_-dependence of the twisted double-bilayer WSe_2_, which is not seen in the natural 4L structure. Under strong doping of holes, larger fields are required to induce the same Stark shift of the intersubband transitions, presumably due to Coulombic screening effects. As shown in twisted bilayer WSe_2_, competing electronic states and layer polarization can emerge at different filling factors and are controlled by out-of-plane electric fields.^28,29^ We tentatively attribute this difference in doping dependence between natural and twisted four-layer structures to the effect of the moiré superlattice on localizing charges and band foldings.^11,29,30^ To understand the detailed mechanism responsible for this effect, further experiments and theory are called for, going beyond the scope of the present work.

To establish the generality of electrically tunable intersubband transitions in transition-metal dichalcogenide multilayers, we fabricated dual-gate devices with natural 2H-stacked five-, six-, and seven-layer WSe_2_ and measured the Stark shift of the intersubband transitions as shown in Figure 4. Panels a, e, i show intersubband transitions for resident electrons and panels c, g, k for resident holes. The experimental results align remarkably well with the calculations of intersubband transitions for CB (Q) shown in panels b, f ,j and for VB­(Γ) in panels d, h, l. Interestingly, several nodes appear in the intensity of the measured electronic Raman spectra at certain field strengths in Figure 4g, k, j; i.e., the Raman intensity changes nonmonotonically with electric field strength in both electron and hole doping regimes. While the complete mechanism of electronic Raman scattering involves higher-order processes in reciprocal space,^9,31^ the real-space wave function overlap between initial and final electronic states can also significantly influence the Raman scattering efficiency. Our analysis of wave function delocalization across different layers as a function of the electric field (Supplementary Figure 10) reveals interesting nonmonotonic field dependencies in both the Q and Γ valley subbands, which may provide a possible explanation for the nonmonotonic changes in Raman intensity observed experimentally. As marked by vertical arrows in Supplementary Figure 10, these nonmonotonic features are associated with crossings of the layer character. Notably, our calculations show that these crossings occur at progressively lower field strengths as the number of layers increases, consistent with the experimental observation of nodes in the Raman intensity shown in Figure 4.

**Figure 4:**
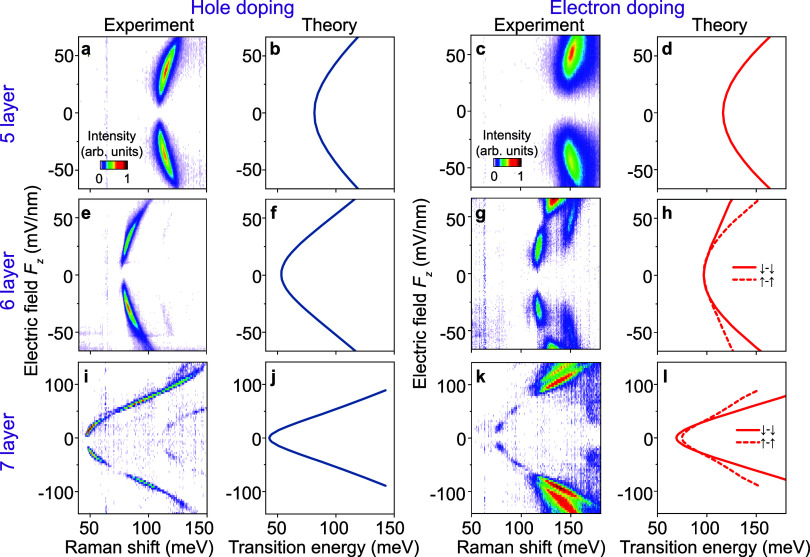
Electrically tunable intersubband transitions in five-, six-, and seven-layer WSe_2_. Experimental Stokes Raman spectra and theoretical intersubband transition energies as a function of the out-of-plane electric field for hole doping (left) and electron doping (right) for natural 2H five-layer (a–d), six-layer (e–h), and seven-layer (i–l) WSe_2_. The experimental data were collected at a doping of |*n*| = 4.7 × 10^12^ cm^–2^ for five and six layers, and at |*n*| = 4 × 10^12^ cm^–2^ for seven layers.

## Discussion

We fit the experimental and theoretical Stark shifts of intersubband transitions in Figures 1–4 to the functionality *E*(*F*_z_) = *E*_0_ – μ­(*F*_z_ – *F*_z0_) + α­(*F*_z_ – *F*_z0_)^2^/2, where *E*_0_ is the intersubband transition energy at field zero, μ is the first-order Stark shift, α is the second-order Stark shift, and *F*_z0_ accounts for asymmetric screening in the experimental data.^24^ Since these lowest-order terms dominate the field dependence of the transition energy for small electric fields, μ can be interpreted as an effective dipole moment and α as an effective polarizability. For an odd number of layers, the effective dipole moments for intersubband transitions at both CB­(Q) and VB­(Γ) must be zero due to the inversion symmetry in the out-of-plane direction. For an even number of layers, the effective dipole moments for transitions at VB­(Γ) must also be zero due to the symmetry argument detailed in Supplementary Note 4.3. To fit the experimental data, linear contributions to the Stark shift are thus only considered for Q valley transitions in four-layer and six-layer samples to prevent competition with the small linear term α*F*_z0_ (cf. Supplementary Note 3). In these cases, the two Stark-shift parabolas arising from the two nondegenerate spin-conserved intersubband transitions (cf. Figure 2) are fitted using the same absolute value |μ| but with opposite signs. A full description of the fit procedure, comparison plots, and tabulated fit results for both experimental and theoretical data are shown in Supporting Information Notes 3 and 5.2. For transitions at VB­(K) in the three-layer system, the vanishing Raman intensity near zero electric field made parabolic fits to experimental data unreliable. In this case, we only report an estimate of the zero-field transition energy (cf. Supplementary Figure 13). For systems with more than three layers, no experimental Raman signal could be attributed to VB­(K) transitions, likely due to a lack of resident holes in the K valley. We summarize the layer number dependence of *E*_0_, |μ|, and α for both electron doping (top panels) and hole doping (bottom panels) in Figure 5. The *E*_0_ values for intersubband transitions (solid spheres) in panel a show a clear decrease with increasing layer number, matching well with the predictions of theory (open circles and triangles) in both the CB­(Q) and VB­(Γ) valleys. As the in-plane momentum at the Γ valley is zero, the intersubband transition energies can be described by a modified infinite square-well model:^5^ $E_{1|N}-E_{2|N}=\frac{3\pi^{2}\hbar^{2}}{2m_{z}d^{2}{\left(N+2\nu \right)}^{2}}$ , where ℏ is Planck’s constant, *m*_z_ is the out-of-plane effective carrier mass in the bulk, *d* is the interlayer distance, *N* is the number of layers, and ν is a phenomenological parameter. We fit the model based on our experimental results and obtain *m*_z_ = 1.03*m*_e_ for the out-of-plane effective mass at the Γ valley, assuming an interlayer distance of *d*_W–W_ = 6.477 Å.^5^ This value of *m*_z_ is close to the prediction of DFT of 1.08*m*_e_.^5^ Furthermore, our extracted *E*_0_ values are consistent with the only previously reported experimental values for intersubband transitions in WSe_2_ from s-SNOM measurements^5^ for four- and five-layer samples, differing by less than 10 meV as seen in Figure 5.

**Figure 5:**
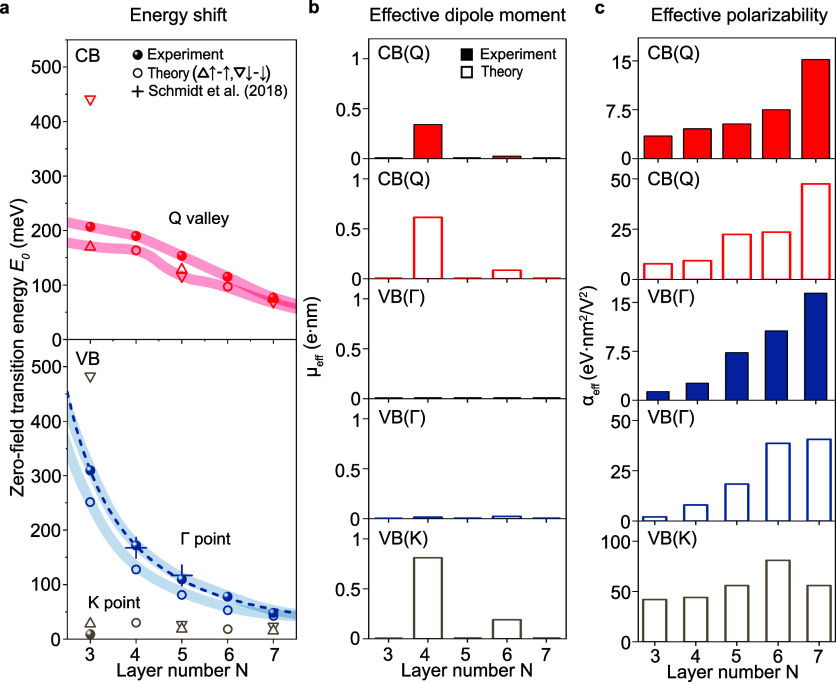
Layer-number dependence of intersubband transitions. (a) Intersubband transition energies obtained by fitting the experimental data (solid spheres) for natural 2H-stacked WSe_2_ crystals of 3 to 7 layers. Thick, transparent lines are guides for the eye. The dashed line indicates a fit of the experimental VB­(Γ) data to a modified infinite-square-well model. The corresponding DFT intersubband transition energies are shown as open circles or triangles if the transitions for the two spin orientations are nondegenerate. For comparison, experimental data by Schmidt *et al*.^5^ for VB­(Γ) are shown as crosses. (b–c) Effective dipole moments and polarizabilities extracted by fitting a parabolic model to the Stark shift of the lowest-energy zero-field intersubband transitions. The top panels are for resident electrons at CB­(Q), and the bottom panels are for holes at VB­(Γ). Note that experimental data for VB­(K) were insufficient for parabolic fitting, and that for fits to experimental data, the effective dipole moment is set to zero except for the case of CB­(Q) in the four- and six-layer samples.

## Conclusions

In summary, we have demonstrated how intersubband transitions in vdW quantum wells—natural multilayer WSe_2_—can be probed directly through inelastic light scattering at visible wavelengths. The technique is highly sensitive and reveals intersubband transitions in several band minima and across a wide range of layer numbers. These intersubband transitions are widely tunable by an electric field, taking advantage of the small intrinsic thickness of few-layer vdW semiconductors and their large effective polarizabilities. Twisted double-bilayer WSe_2_ shows an unexpected doping dependence of the electrical tunability of intersubband transitions, indicating an additional degree of freedom for controlling these transitions in vdW quantum wells. Intersubband transitions can potentially be used to design tunable narrow-band IR photodetectors.^32^ As the detection energy is tunable by an electric field, the design of an atomically thin IR spectrometer without the need for dispersive elements appears feasible. As a spectroscopic tool at visible wavelengths in the blue spectral region, far-field Raman scattering should also provide sufficient spatial resolution for imaging applications, which would prove particularly useful in measuring inhomogeneities in electric-field strengths in double-gated device structures. We expect that the phenomenon can ultimately be extended into the subdiffraction regime by near-field enhanced Raman scattering techniques.

## Methods

### Device Fabrication and Characterization

Flakes of hexagonal Boron Nitride (hBN), graphite, and WSe_2_ were mechanically exfoliated from bulk crystals on Si/SiO_2_ substrates. Flakes were selected through characterization using an optical microscope and an atomic force microscope (AFM). The layer number of WSe_2_ flakes was precisely determined through a combination of AFM measurements, optical contrast analysis, and optical second-harmonic generation spectroscopy. The dual-gate devices were assembled using a layer-by-layer dry transfer technique. The dual-gate device of twisted double-bilayer WSe_2_ was fabricated using a “tear-and-stack” technique. This process involved tearing a large bilayer WSe_2_ flake, picking up one section and then restacking it onto the remaining portion with a precisely controlled twist angle, achieved using a high-precision rotation stage. Electrical contacts were established by patterning Cr/Au electrodes using standard electron beam lithography, followed by metal evaporation.

### Optical Spectroscopy

Optical measurements were performed using a confocal microscope setup in backscattering geometry with a cryogenic reflective objective with a numerical aperture of 0.5. Samples were loaded into a closed-cycle cryostat (Attocube Systems, attoDRY2100) and measured in low-pressure He exchange gas at 1.8 K, unless otherwise stated. Several continuous-wave lasers with different wavelengths were used for excitation: a 488 nm diode laser (Coherent, Sapphire 488 SF NX), a 532 nm diode-pumped solid-state laser (Spectra, Millennia eV), and a tunable, frequency-doubled Ti:sapphire laser (Sirah, Matisse CR and WaveTrain 2). Polarization-resolved measurements were performed with a set of broadband half-wave and quarter-wave plates and linear polarizers. A room-temperature objective with a numerical aperture of 0.6 (Olympus, LUCPLFLN, 40×) was used to measure the 5L and 6L samples in a coldfinger cryostat (CryoVac, Konti Micro). In both cases, the emission was dispersed by ruled gratings (600 grooves mm^–1^) in commercial spectrometers (Andor and Princeton Instruments) and recorded by charge-coupled device (CCD) cameras (Andor Newton 920 or Princeton Instruments PIXIS 100).

### Doping Density and Electric Field

The doping density and out-of-plane electric field in the multilayer crystals are determined from the applied gate voltages based on a parallel-plate capacitor model.^12,13^ Using the geometric capacitance per unit area *C*_t,b_ = ε_0_ε_hBN_/*d*_hBN_, the charge-carrier density is calculated as *n* = (*C*_t_ *V*_t_ + *C*_b_ *V*_b_)/*e* and the electric field as $F_{z}=\varepsilon_{\mathrm{hBN}}\left(V_{t}-V_{b}\right)/\varepsilon_{{\mathrm{WSe}}_{2}}\left(d_{hBN,t}+d_{hBN,b}\right)$ , where *d*_hBN,t_ and *d*_hBN,b_ are the thicknesses of the top and bottom hBN layers as measured by AFM, *V*_t_ and *V*_b_ are the top and bottom gate voltages, and *ε*_hBN_ = 3.4 (ref 33) and $\varepsilon_{{\mathrm{WSe}}_{2}}=7.2$ (ref 34) are the dielectric constants of hBN and WSe_2_, respectively.

### DFT Calculations

The electronic properties of natural WSe_2_ multilayers under an electric field were calculated with the all-electron full-potential linearized augmented plane-wave method as implemented in the Wien2k package.^35^ We used the Perdew–Burke–Ernzerhof^36^ exchange-correlation functional with vdW interactions accounted for by the D3 correction.^37^ The wave function expansion into atomic spheres takes into account orbital quantum numbers of up to 10, and the plane-wave cutoff multiplied by the smallest atomic radii was set to 8. Spin–orbit coupling was included fully relativistically for core electrons, while valence electrons were treated within a second variational procedure^38^ with the scalar-relativistic wave functions calculated in an energy window up to 1.9 Ry. Self-consistency was achieved using a two-dimensional Monkhorst–Pack k-grid with 15 × 15 points and the convergence criteria of 10^–6^ e for the charge and 10^–6^ Ry for the energy. We considered values of 3.334 Å for the lattice parameter, 3.330 Å for the monolayer thickness, 3.073 Å for the interlayer distance (all taken from ref 39), and a vacuum space of 60 Å. The out-of-plane electric field was modeled with a zigzag potential added to the exchange-correlation functional.^40^ We expanded the electric field potential with 40 Fourier coefficients with the multilayer system centralized at 1/4 of the unit cell in the out-of-plane direction. This approach successfully reproduces the observed trends in natural bilayer WSe_2_ ^13^ and MoSe_2_/WSe_2_ heterostructures.^24^ The input electrostatic potential energy, *V*_0_, is converted to electric field as *F*_z_ = $\frac{1}{\varepsilon_{{\mathrm{WSe}}_{2}}}\frac{2V_{0}}{c}$ , in which *c* is the size of the supercell in the out-of-plane direction, and we incorporate the dielectric constant of WSe_2_ to be consistent with the experimental definition of the electric field. Further details on the theory and complementary plots are available in Supporting Information 4 and 5.

## Supplementary Material



## Notes

Supplementary Figures 1–27 and Supplementary Tables 1–2 referred to in the main text showing the doping dependence of electronic Raman scattering from 3L and 4L samples, helicity-resolved Raman scattering of 3L and 4L samples, the wavelength dependence of Raman scattering from 3L and 4L samples, the temperature dependence of Raman scattering from the 4L sample, the laser-power dependence of Raman signals from 4L WSe_2_, extraction of intersubband transition energies and their first- and second-order Stark shifts, results from DFT calculations with and without an out-of-plane electric field, and micrographs of the twisted- and natural-multilayer WSe_2_ device structures (PDF)
