# Supplementary material for: Electrical Control of Intersubband Transitions in Few-Layer WSe2 Multivalley Quantum Wells Probed by Electronic Raman Scattering
Source: ACS Nano. 2026 Jan 24;20(5):4018–26. doi: 10.1021/acsnano.5c08378 (PMC12895561; doi:10.1021/acsnano.5c08378)
Supplement: Supplementary file 1 [file nn5c08378_si_001.pdf]

Supporting Information for Publication

Electrical control of intersubband transitions in few-layer WSe_2 multi-valley quantum wells probed by electronic Raman scattering

Philipp Wutz[1,9], Yinong Zhang[2,9], Felix Hofmann[1,9], Paulo E. Faria Junior[3,4,5]*, Yao Lu[6], Philip Soul[1], Yu-Han Bao[6], Kenji Watanabe[7], Takashi Taniguchi[8], Jaroslav Fabian[3], Sebastian Bange[1], John M. Lupton[1]*, Kai-Qiang Lin[1,6]*

[1]Institute for Experimental and Applied Physics, University of Regensburg, 93053 Regensburg, Germany.

[2]Department of Physics, University of Washington, Seattle, WA 98195-1560, USA.

[3]Institute for Theoretical Physics, University of Regensburg, 93053 Regensburg, Germany.

[4]Department of Physics, University of Central Florida, Orlando, Florida 32816, USA.

[5]Department of Electrical and Computer Engineering, University of Central Florida, Orlando, Florida 32816, USA.

[6]State Key Laboratory of Physical Chemistry of Solid Surfaces, College of Chemistry and Chemical Engineering, Xiamen University, 361005 Xiamen, China.

[7]Research Center for Electronic and Optical Materials, National Institute for Materials Science, 1-1 Namiki, Tsukuba 305-0044, Japan.

[8]Research Center for Materials Nanoarchitectonics, National Institute for Materials Science, 1-1 Namiki, Tsukuba 305-0044, Japan.

[9]These authors contributed equally.

*E-mail: paulo@ucf.edu; john.lupton@ur.de; kaiqiang.lin@xmu.edu.cn

Contents

Supplementary Note 1: Extended figures 2

Supplementary Note 2: Power dependence of electronic Raman signals from 4L WSe_2 12

Supplementary Note 3: Extracting intersubband transition energies and their first- and second-order Stark shifts 13

Supplementary Note 4: DFT calculations without an out-of-plane electric field 17

Supplementary Note 5: DFT calculations with an out-of-plane electric field 22

Supplementary Note 6: Micrographs of the twisted- and natural-multilayer WSe_2 devices 30

Supplementary Figs. 1-27

Supplementary Tables 1-2

Supplementary Note 1: Extended Figures

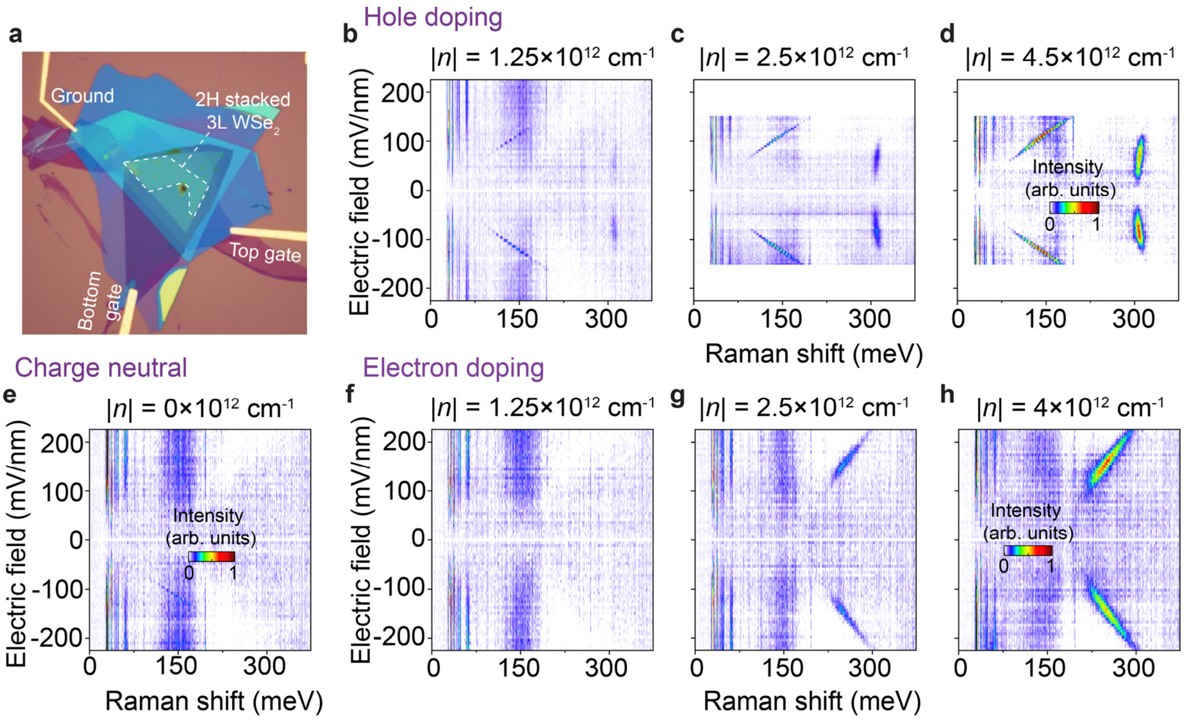

Supplementary Figure 1 | Raman scattering of trilayer WSe_2 as a function of out-of-plane electric field at different doping densities. a, Micrograph of the trilayer WSe_2 transistor device, in white-light reflection geometry. Top and bottom hBN layer thicknesses are 17.3 nm and 11.8 nm, respectively, as measured by atomic force microscopy. **b-h,** Raman scattering intensity as function of out-of-plane electric field and Stokes Raman shift for hole doping (b-d**)**, at the charge neutrality point (e), and for electron doping (f-h). All Raman spectra were excited with a 488-nm laser, and are shown after subtraction of the of the response at zero electric field.

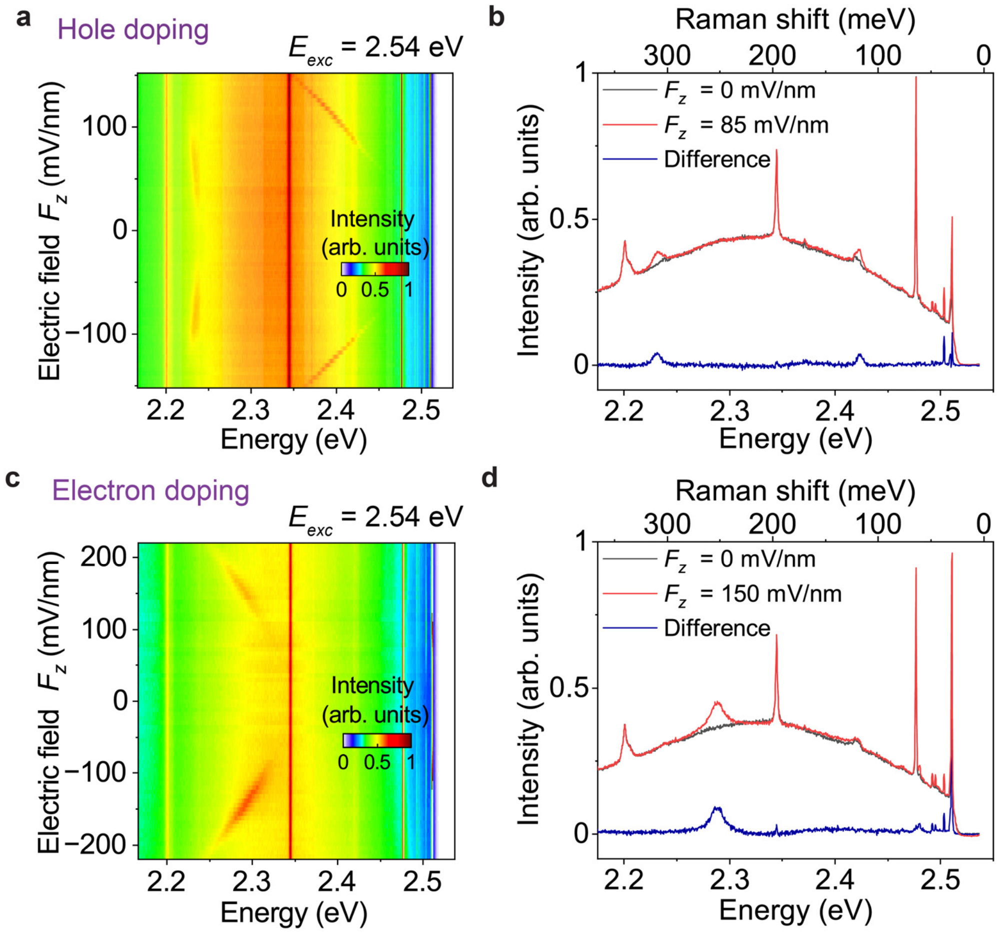

Supplementary Figure 2 | Raw Raman spectra of the trilayer WSe_2 device, without the zero-field subtraction. a, Raman scattering intensity of trilayer WSe_2 as function of F_z and photon energy at a hole doping of $|n| = 4.5 \times 10^{12}\mathrm{cm}^{-2}$. **b,** Corresponding Raman spectra at F_z = 0 mV/nm (grey) and F_z = 85 mV/nm (red), along with their difference (blue). **c,** Raman scattering intensity as function of F_z and photon energy at an electron doping of $|n| = 4 \times 10^{12}\mathrm{cm}^{-2}$. **d,** Corresponding Raman spectra at F_z = 0 mV/nm (grey) and F_z = 150 mV/nm (red), and the difference between the two (blue). All Raman spectra were excited with a 488-nm laser.

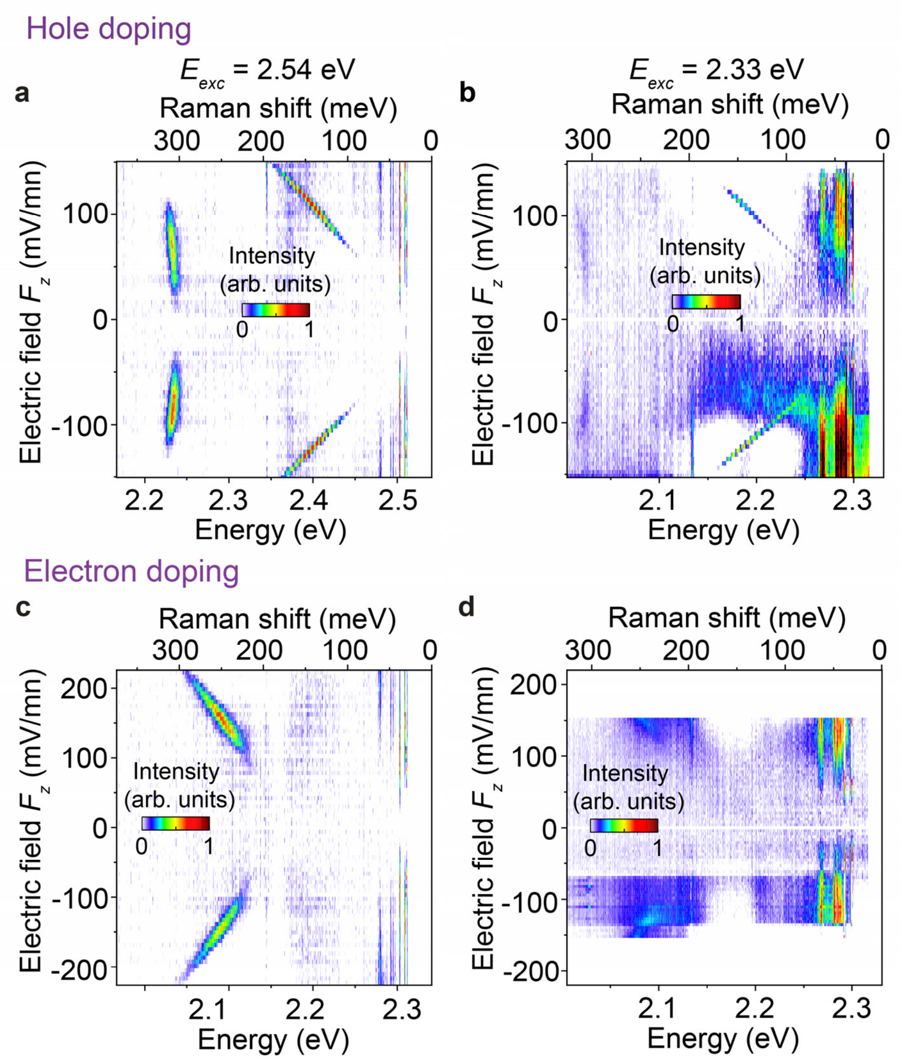

Supplementary Figure 3 | Raman spectra of trilayer WSe_2 at different excitation wavelengths. a,c, Raman spectra of trilayer WSe_2 as a function of out-of-plane electric field (F_z) under 488 nm (2.54 eV) excitation for hole (a) and electron (c) doping, plotted against both photon energy and Stokes Raman shift. **b,d,** Raman spectra of trilayer WSe_2 as a function of F_z under 532 nm (2.33 eV) excitation for hole (b) and electron (d) doping. All spectra are corrected by subtracting the corresponding zero-field spectra.

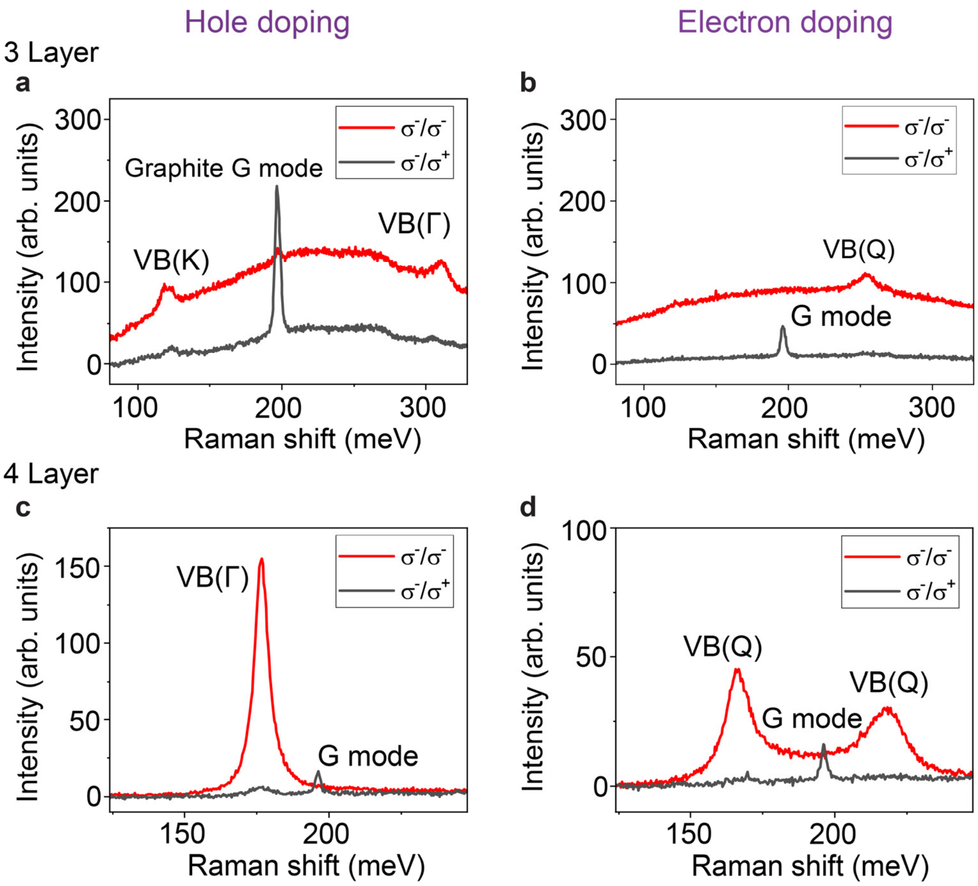

Supplementary Figure 4 | Helicity-resolved Stokes Raman spectra of natural three- and four-layer WSe_2. a, Helicity-resolved Raman spectra of trilayer WSe_2 with electron doping of $|n| = 4 \times 10^{12}$ cm^{-2} and an electric field of -151 mV/nm. **b,** Helicity-resolved Raman spectra of trilayer WSe_2 with a hole doping of $|n| = 4.5 \times 10^{12}$ cm^{-2} and an electric field of 85 mV/nm **c,d,** Helicity-resolved Raman spectra of four-layer WSe_2 at an electron and hole doping of $|n| = 4 \times 10^{12}$ cm^{-2} and an electric field of 63 mV/nm. The Raman signal from the Q, Γ and K valleys can only be detected in the copolarized channel. The G mode from the graphite serves as a reference. All Raman spectra were excited with a 488-nm laser.

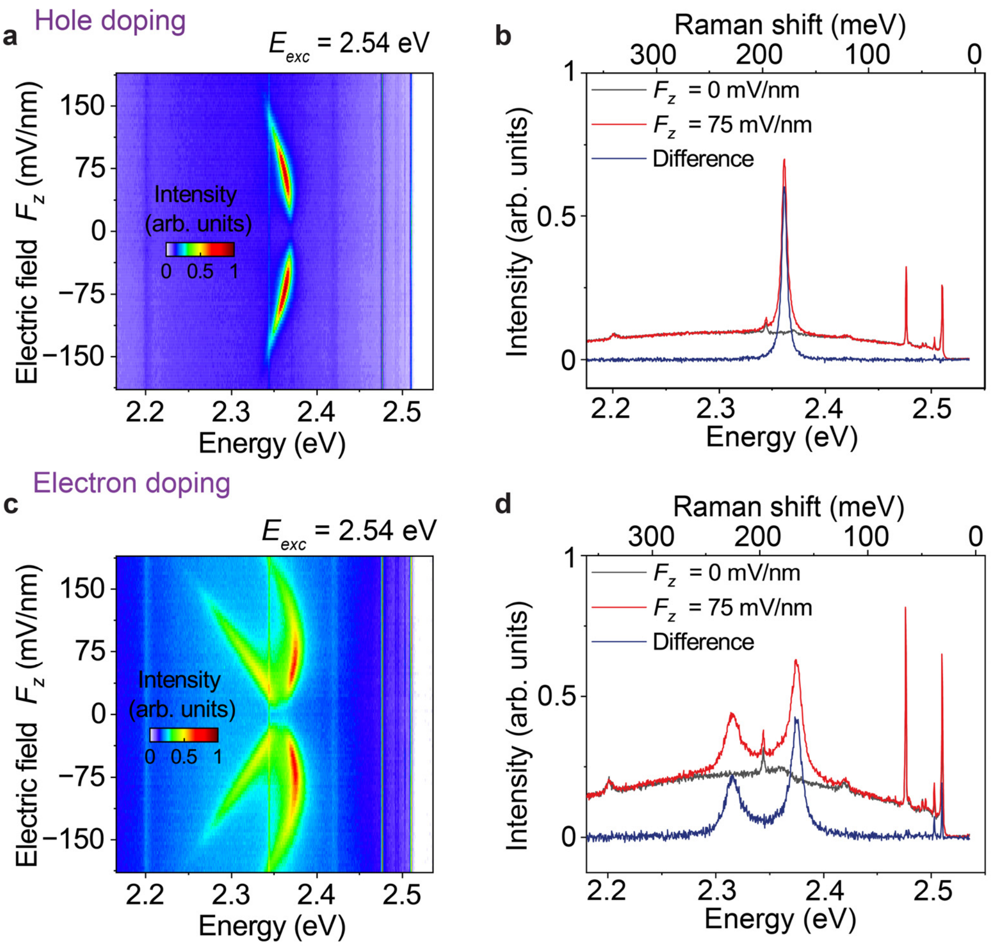

Supplementary Figure 5| Raw Raman spectra of a four-layer WSe_2 device. a, Raman scattering intensity from a trilayer WSe_2 device as function of F_z and photon energy at a hole doping of $|n| = 4 \times 10^{12} \mathrm{cm}^{-2}$, under 488 nm (2.54 eV) excitation. **b,** Corresponding Raman spectra at F_z = 0 mV/nm (grey) and F_z = 75 mV/nm (red), and the difference between the two (blue). **c** Raman scattering intensity at an electron doping of $|n| = 4 \times 10^{12} \mathrm{cm}^{-2}$, under 488 nm (2.54 eV) excitation. **d,** Corresponding Raman spectra at F_z = 0 mV/nm (grey) and F_z = 75 mV/nm (red), and the difference between the two (blue).

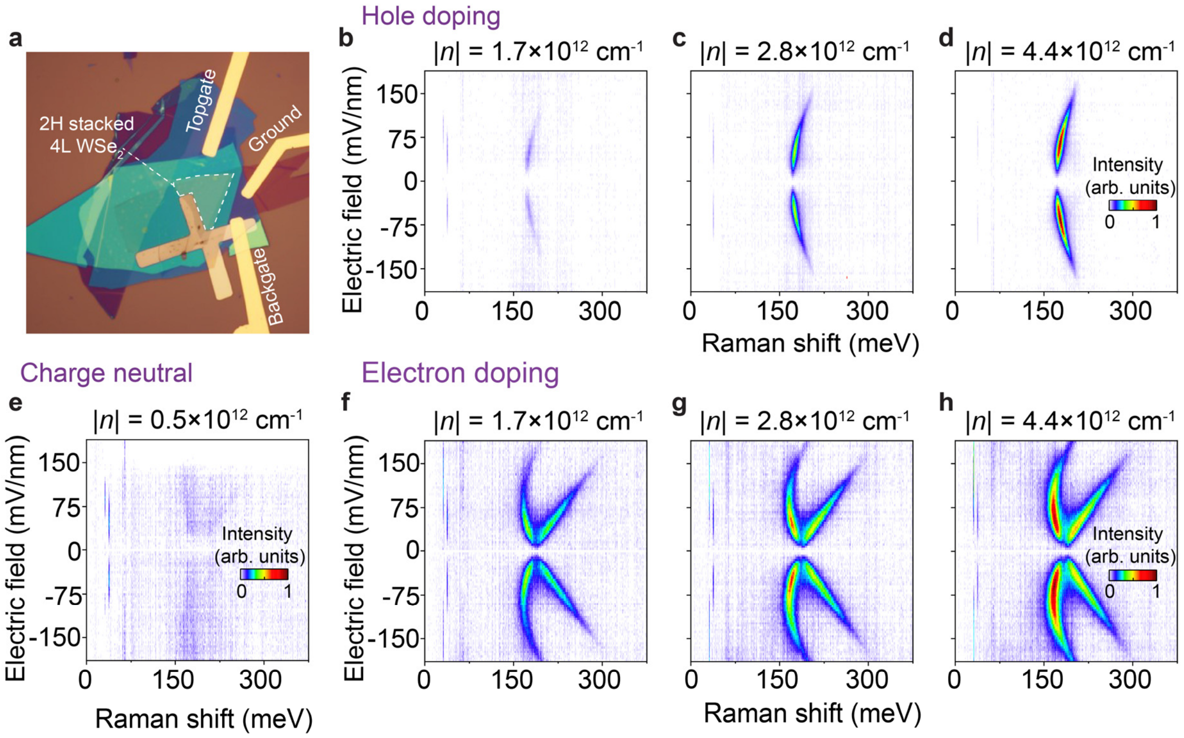

Supplementary Figure 6 | Raman scattering of four-layer WSe_2 as a function of out-of-plane electric field at different doping densities. a, Micrograph of the 2H-stacking natural four-layer WSe_2 transistor device. The top and bottom hBN layer thickness is 17 nm, as characterized by atomic force microscopy. **b-h,** Raman scattering as a function of out-of-plane electric field for hole doping (b-d), at the charge neutrality point (e), and for electron doping (f-h). All spectra are corrected by subtracting the corresponding spectra at zero field. All Raman spectra were excited with a 488-nm laser.

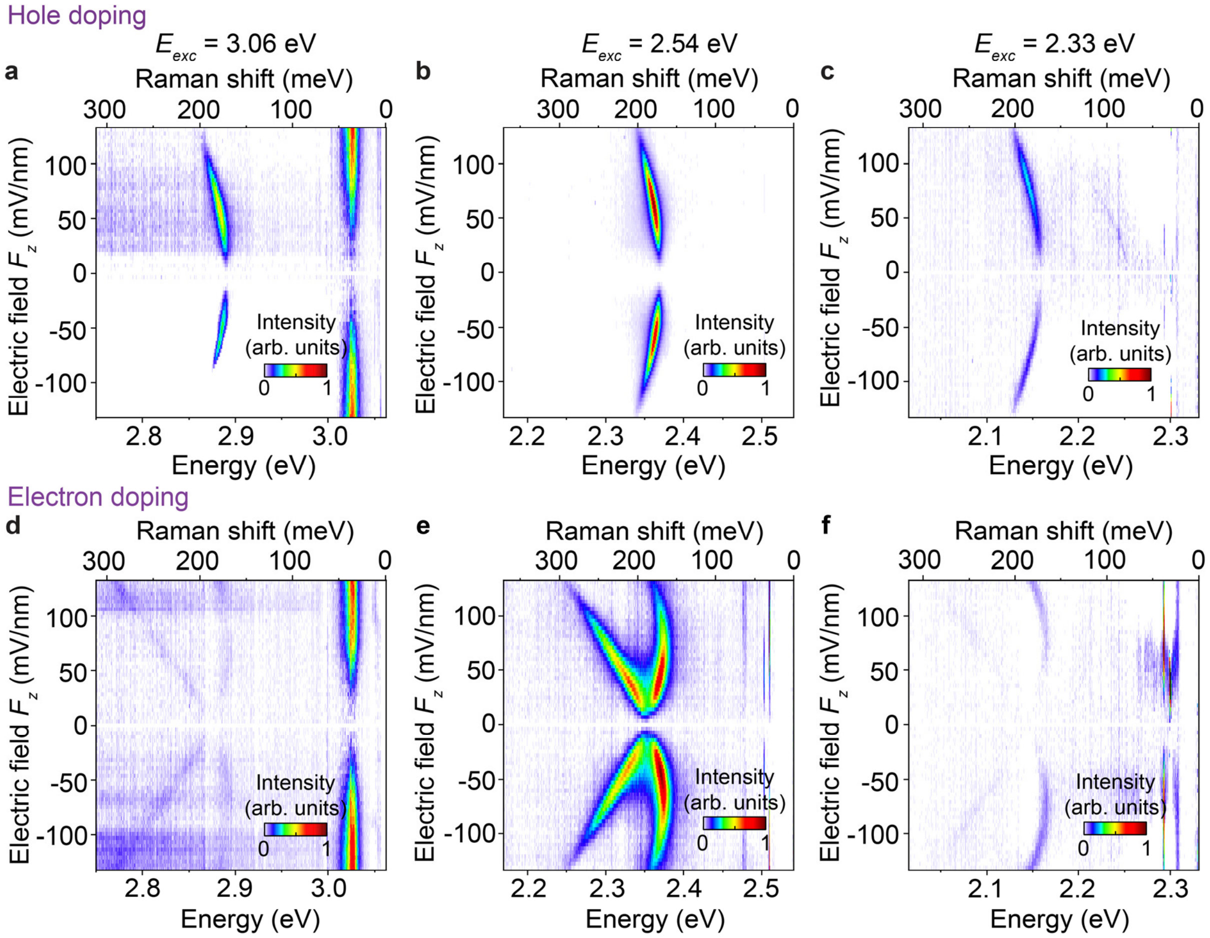

Supplementary Figure 7 | Raman spectra of four-layer WSe_2 for different excitation wavelengths. a-c, Raman spectra of four-layer WSe_2 as a function of F_z for hole doping under 405 nm (3.06 eV) excitation (a), 488 nm (2.54 eV) excitation (b), and 532 nm (2.33 eV) excitation (c). **d-f,** Raman spectra of four-layer WSe_2 as a function of F_z for electron doping under 405 nm (3.06 eV) excitation (d), for 488 nm (2.54 eV) excitation (e), and 532 nm (2.33 eV) excitation (f). All spectra were corrected by subtracting the corresponding spectra at zero field.

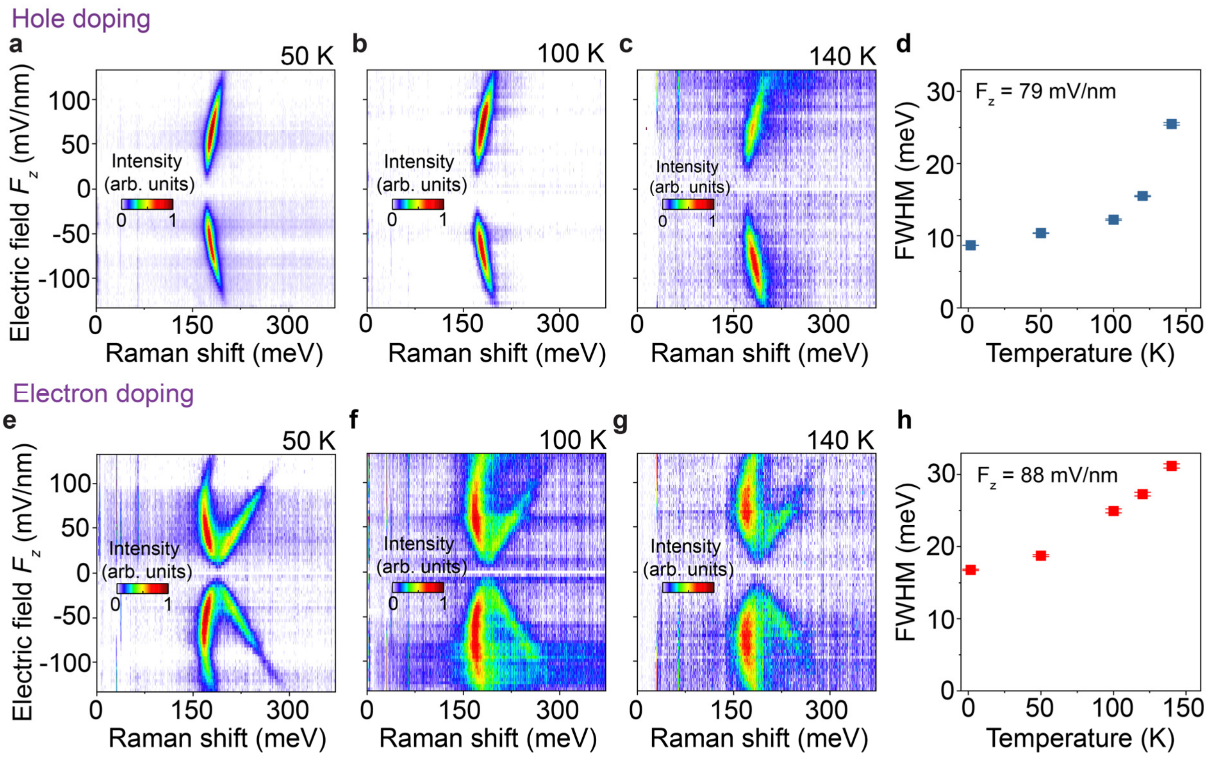

Supplementary Figure 8 | Raman spectra of four-layer WSe_2 as a function of the out-of-plane electric field for hole (a-c) and electron (e-g) doping at different temperatures and their respective temperature-dependent full width at half maximum (FWHM) for a constant electric field for hole (d) and electron (h) doping. All Raman spectra were excited with a 488-nm laser and are shown after subtraction of the corresponding spectra at zero field. The FWHM was extracted by fitting the corrected data with a Gaussian.

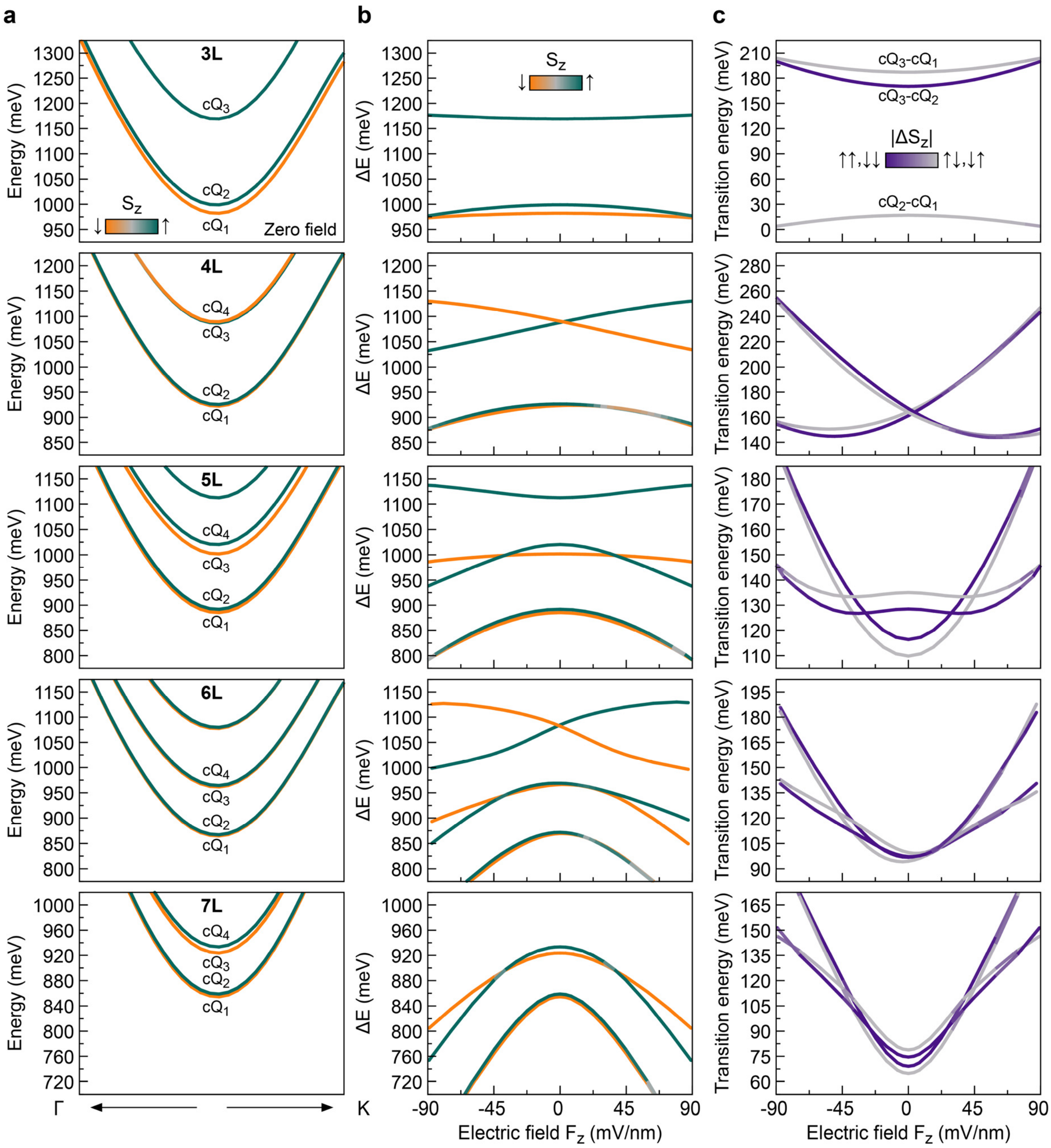

Supplementary Figure 9 | Intersubband transitions of the conduction band at the Q valley for different numbers of layers. a, A magnified view of the band structure under zero electric field is displayed, where the color coding indicates the spin expectation value. **b,** Evolution of the band minima in response to varying electric-field strength, with colors again representing the spin expectation value. **c,** Progression of different intersubband transitions as the electric field changes, using color coding to represent the absolute net spin of each transition.

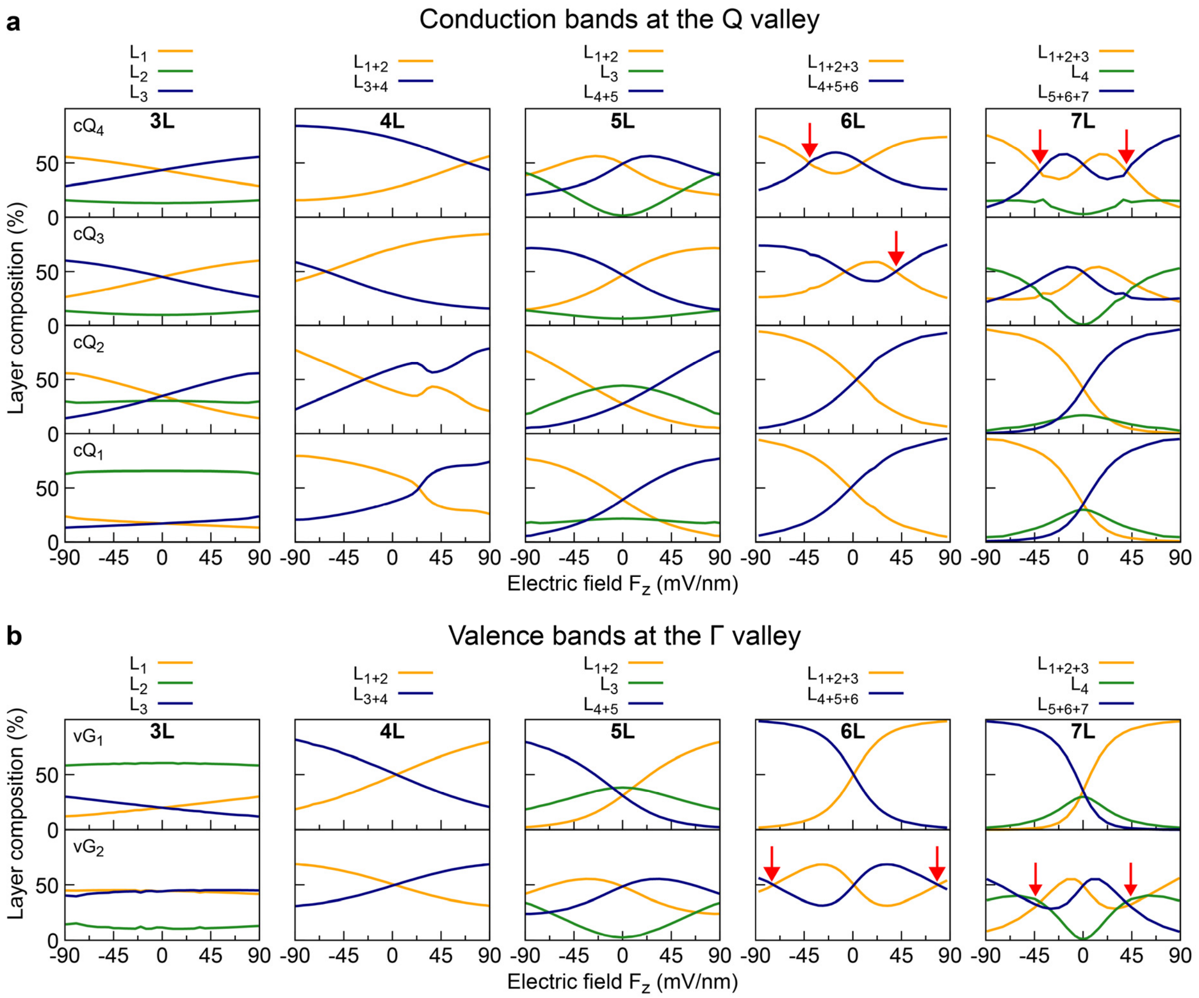

Supplementary Figure 10 | Layer decomposition of the wavefunction for conduction bands at the Q valley (a) and for valence bands at the Γ valley (b) as a function of electric field. Non-monotonic features appear in higher-energy subbands for both Q and Γ valleys in 6L and 7L systems. Red vertical arrows mark crossings of the layer character.

Supplementary Note 2: Power dependence of electronic Raman signals from 4L WSe_2

The excitation-power dependence was measured for the natural four-layer WSe_2 sample as its electronic Raman signal was the brightest. The scattering spectra are depicted in Supplementary Figure 1 a,b for different excitation powers at a constant electric field of $F_z = 63$ mV/nm for hole and $F_z = 81$ mV/nm for electron doping, at a doping level of $|n| = 4 \times 10^{12}$ cm^{-2}. All spectra are corrected by subtracting the spectrum at zero electric field. The integrated intensities are depicted in Supplementary Figure 11 c,d as a function of excitation power. The integrated signals are consistent with a linear dependence between scattering intensity and laser power.

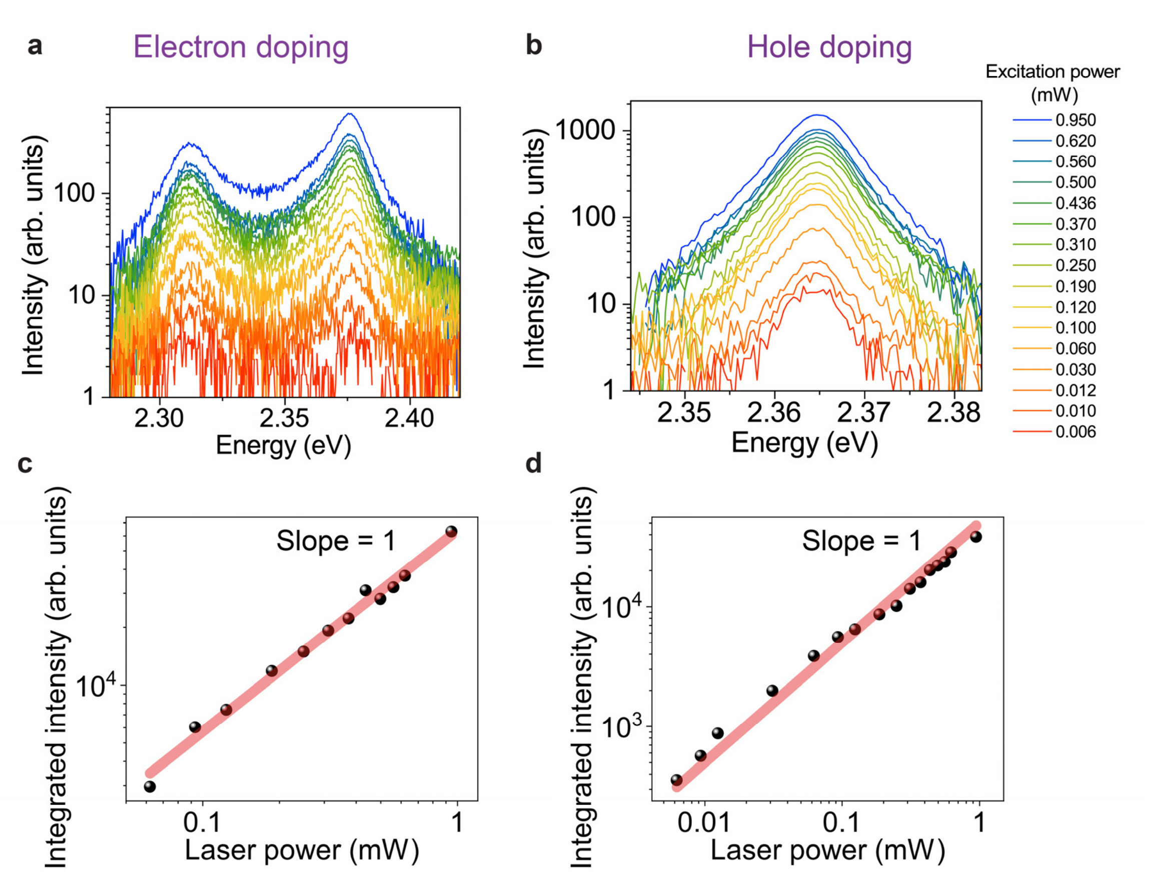

Supplementary Figure 11 | a,b, Power dependence of the Raman signal at a fixed electric field and doping level for electron (a) and hole (b) doping. **c,d,** The corresponding spectrally integrated signal strengths as a function of laser power on a double-logarithmic scale. The red lines indicate a slope of unity, i.e. a direct proportionality. All Raman spectra were excited with a 488-nm laser, and are shown after subtraction of the response at zero electric field.

Supplementary Note 3: Extracting intersubband transition energies and their first- and second-order Stark shifts

To determine the energy of the intersubband transitions, we fitted each observed Raman-scattering spectral peak with a Gaussian to extract the peak position and subtracted its energy from the laser photon energy of 2.541 eV. The electric-field dependence of these transition energies was then fitted by a second-order polynomial to quantify the first and second-order Stark shift and to extract the transition energy at zero electric field. The polynomial fits were guided by considering the structural symmetries of the different samples, as detailed in Supplementary Note 4.3. In short, linear contributions to the Stark shift are only considered for the CB(Q) valley, and only in systems with an even number of layers. In this case, the spatial inversion symmetry causes a spin-flipped replica of the transition to appear upon switching of the out-of-plane electric field direction. In addition, it is experimentally impossible to exclude asymmetric screening effects when applying electric fields from the top and bottom gates. It is thus necessary to allow for an offset F_{z0} when using electric-field values F calculated directly from experimental gate voltages. For Q valley transitions in four-layer and six-layer samples, we thus used the polynomial function

$$E(F_z) = E_0 - \mu(F_z - F_{z0}) + \frac{\alpha}{2}(F_z - F_{z0})^2 \qquad (1)$$

for fitting the transition energy E as function of the calculated electric fields F_z. Although both αF_{z0} and μ cause linear contributions to the field dependence, consistent parameters can be found by using a single global fit for both positive and negative μ branches. The remaining transitions were fitted by

$$E(F_z) = E_0 + \frac{\alpha}{2}(F_z - F_{z0})^2, \qquad (2)$$

in which case the linear contribution is only due to αF_{z0} and not due to a dipolar contribution. The peak positions and parabolic fits are depicted in Supplementary Figure 12, and the resulting numerical values for all fit parameters are summarized in Supplementary Table 1.

In the three-layer system, second-order polynomial fitting of the intersubband transition energies associated with the VB(K) valley was found to be unreliable, owing to a combination of large Stark shifts and a lack of data points at small electric fields. We thus limit the analysis to determining a lower bound for the zero-field transition energy from linear fits at positive and negative electric fields, as shown in Supplementary Figure 13.

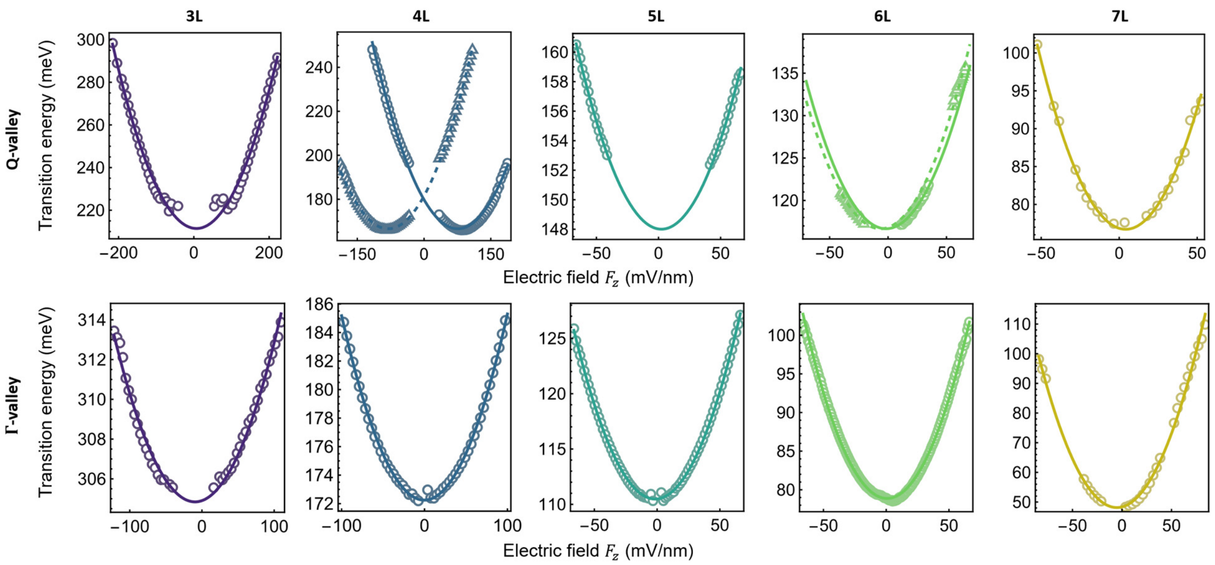

Supplementary Figure 12 | Polynomial fits of the intersubband transition energy as a function of the nominal out-of-plane electric field. Results for the electronic transitions associated with the CB(Q) valley (top row); Results for for electronic transitions associated with the VB(Γ) valley (bottom row). Solid (dashed) fits for 4L and 6L Q valley data correspond to the positive (negative) branches of the linear field dependence.

Sample/Valley	E_0 in meV	F_{z0} in V/nm	$\|\mu\|$ in e · nm	α in eV · nm^2/V^2
3L / VB(Γ)	304.8(1)	-0.0096(5)	-	1.33(3)
3L / CB(Q)	211.4(8)	0.0070(8)	-	3.48(6)
4L / VB(Γ)	172.25(3)	-0.0002(1)	-	2.60(2)
4L / CB(Q)	180.6(2)	-0.0024(3)	0.359(2)	4.59(3)
5L / VB(Γ)	110.48(2)	-0.00156(5)	-	7.28(2)
5L / CB(Q)	148.0(1)	0.0023(1)	-	5.33(6)
6L / VB(Γ)	78.86(4)	0.00072(6)	-	10.63(4)
6L / CB(Q)	116.7(1)	-0.0041(2)	0.017(2)	7.5(1)
7L / VB(Γ)	48.1(4)	-0.0052(4)	-	16.4(2)
7L / CB(Q)	76.7(2)	0.0041(2)	-	15.2(3)

Supplementary Table 1 | Parameters of the polynomial fits for the experimental data on various layered systems: intersubband transition energy E_0 at zero electric field, electric-field offset F_{z0} caused by screening, and first and second-order Stark shift parameters μ (effective dipole moment) and α (effective polarizability). The standard fit error for the last digit of the parameters is stated in parentheses.

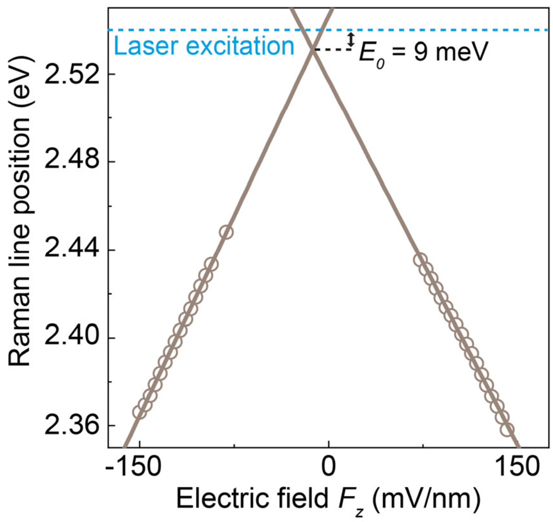

Supplementary Figure 13 | Linear fits of the Raman line position observed for transitions associated with the VB(K) valley for the three-layer sample. The difference between the laser photon energy (blue-dashed line) and intersection of the fits to the branches at positive and negative electric fields provides an estimate of the lower bound of the zero-field transition energy E_0.

Supplementary Note 4: DFT calculations without an out-of-plane electric field

Supplementary Note 4.1 Band structures

The calculated band structures at zero electric field for the different WSe_2 multilayers are presented in Supplementary Fig. 14. Focusing on the regions in which intersubband transitions take place, we show a zoom of the band structures around the conduction bands at the Q valley in Supplementary Fig. 15, the conduction bands at the K point in Supplementary Fig. 16, the valence bands at the Γ point in Supplementary Fig. 17, and the valence bands at the K point in Supplementary Fig. 18.

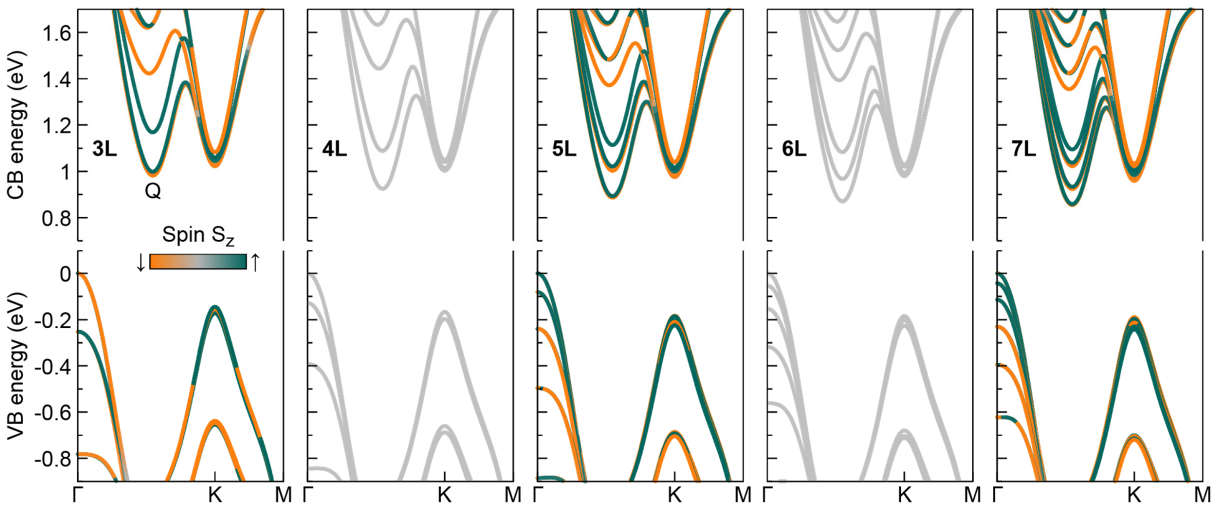

Supplementary Figure 14 | Band structure at zero electric field for 3L, 4L, 5L, 6L, and 7L systems along the high-symmetry direction.

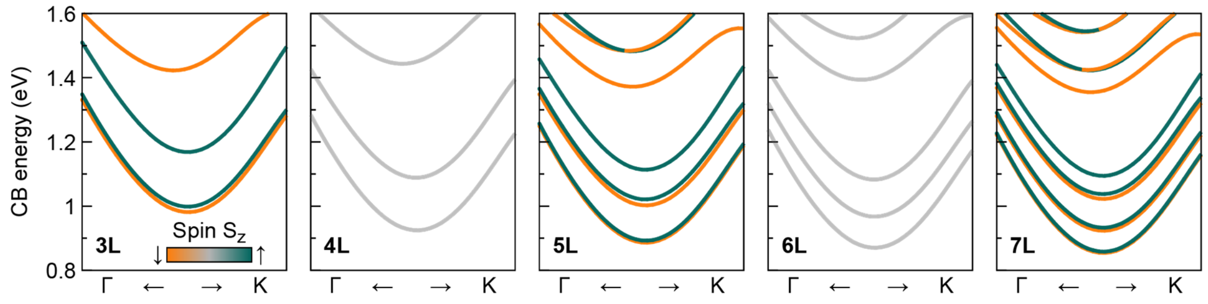

Supplementary Figure 15 | Band structure of the conduction band around the Q valley.

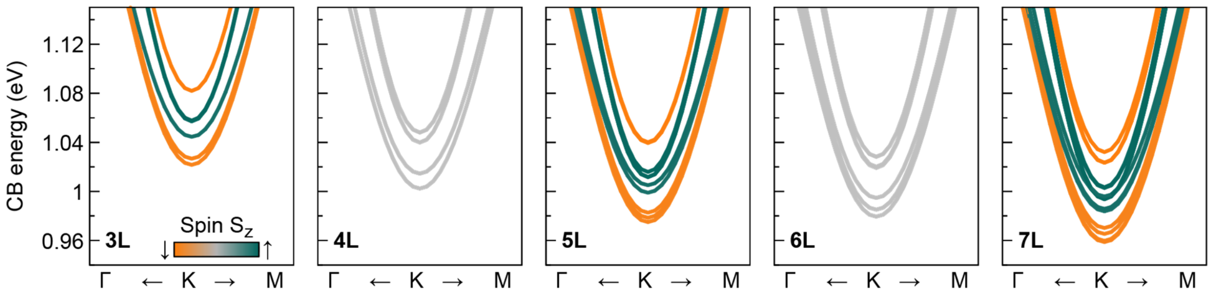

Supplementary Figure 16 | Band structure of the conduction band around the K valley.

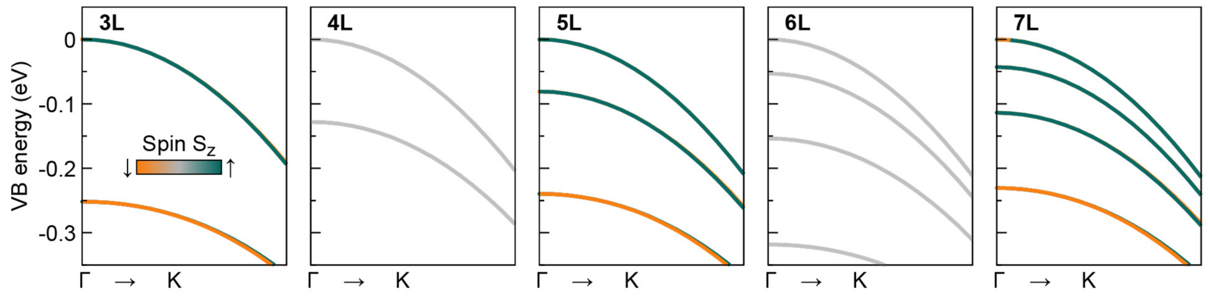

Supplementary Figure 17 | Band structure of the valence band around the Γ valley.

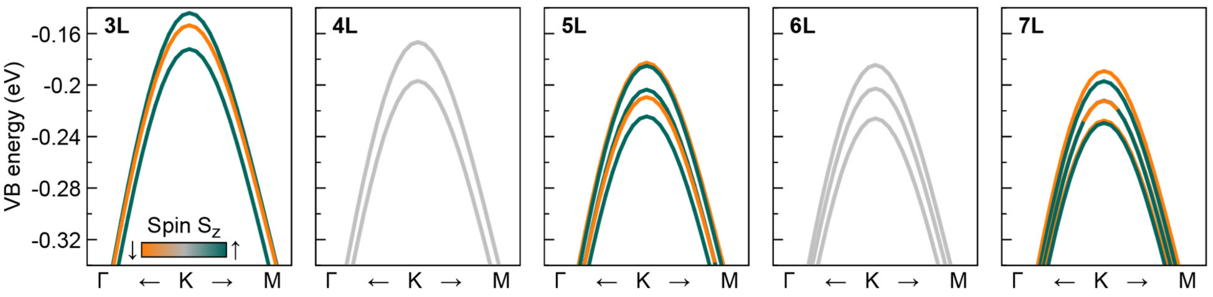

Supplementary Figure 18 | Band structure of the valence band around the K valley.

Supplementary Note 4.2 Energy splitting at zero field

The summary of the layer-number dependence of different intersubband transitions at zero field is given in Supplementary Fig. 19, together with the energy difference between the top valence band at the K valley and the top valence band at the Γ valley. The K→ Γ energy difference increases with layer number, suggesting a suppression of the resident hole in the K valley and thus preventing the detection of intersubband transitions in the VB(K) valley for N > 3.

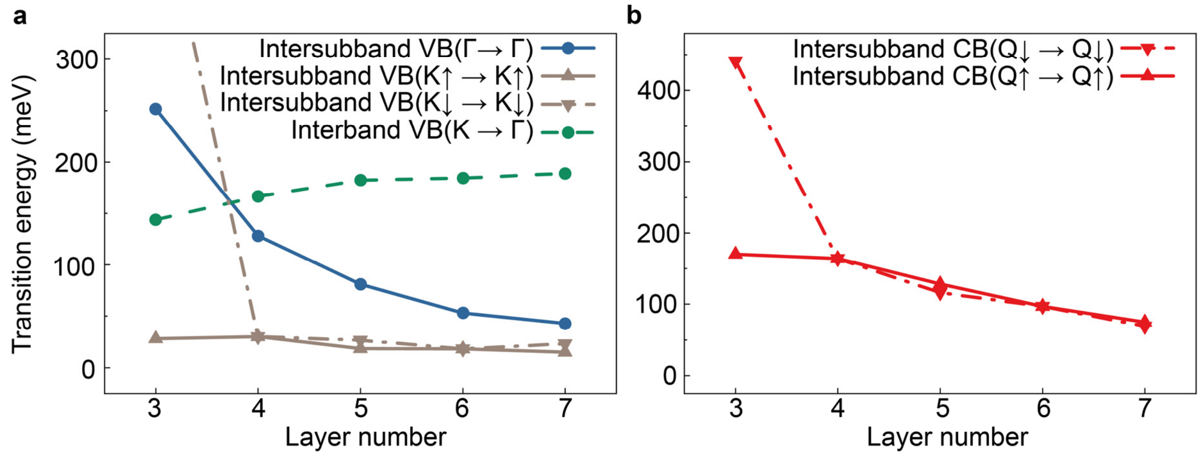

Supplementary Figure 19 | a, Layer number dependence of the lowest-energy intersubband transitions at the VB(Γ) valley (blue), at the VB(K) valley (gray), and the energy difference between the top valence band at the K valley and the Γ valley (green, dashed). The transition energy for spin-down transitions at VB(K) in the three-layer system, mainly controlled by the large VB spin-orbit coupling, is 482.7 meV and beyond the scale of interest for the plot. For reference, cf. Supplementary Fig. 14. **b,** Layer number dependence of the lowest-energy spin-conserved intersubband transitions at the CB(Q) valley.

Supplementary Note 4.3 Electric field effects from perturbation theory and symmetry arguments

From a perspective of perturbation theory, the electric dipole moment and the polarizability are related to the first and second-order energy corrections of the interaction Hamiltonian due to the external electric field, $V_{\mathrm{int}} = -eF_z z$ with $e > 0$. Specifically, the expressions of μ and α for the n-th band at the point $\vec{k}$ can be written as:

$$\mu_{\mathrm{n,\vec{k}}} = \mathrm{e}\langle\psi_{n,\vec{k}}|z|\psi_{n,\vec{k}}\rangle$$
$$\alpha_{n,\vec{k}} = 2e^2 \sum_{m\neq n} \frac{\langle\psi_{n,\vec{k}}|z|\psi_{m,\vec{k}}\rangle\langle\psi_{m,\vec{k}}|z|\psi_{n,\vec{k}}\rangle}{E_n(\vec{k}) - E_m(\vec{k})} \tag{3}$$

The multilayer structures with odd number of layers (symmetry group D_{3h}) contain a horizontal mirror-plane symmetry, which imposes a strong constraint on the electric dipole moment:

$$\sigma_{\mathrm{h}}\psi_{n,\vec{k}} \rightarrow \pm\psi_{n,\vec{k}}$$
$$\sigma_{\mathrm{h}}z \rightarrow -z \tag{4}$$

$$\langle\psi_{n,\vec{k}}|z|\psi_{n,\vec{k}}\rangle \overset{\sigma_{\mathrm{h}}}{\rightarrow} -\langle\psi_{n,\vec{k}}|z|\psi_{n,\vec{k}}\rangle \Rightarrow \langle\psi_{n,\vec{k}}|z|\psi_{n,\vec{k}}\rangle = 0 \tag{5}$$

On the other hand, for systems lacking the horizontal mirror plane, such as the multilayer structures with an even number of layers (symmetry group D_{3d}), this symmetry operation σ_{h} is not present and therefore there is no strong symmetry constraint that imposes $\langle\psi_{n,\vec{k}}|z|\psi_{n,\vec{k}}\rangle = 0$. In this case, one needs to evaluate whether such matrix element exists for the particular band n and wavevector $\vec{k}$. For the Γ point, with a D_{3d} symmetry group, the top valence bands are two-fold degenerate with real (double group) irreducible representations (irreps) $\Gamma_4^{\pm}$ and the operator $z \sim \Gamma_2^-$. A direct product of the energy band irreps yields $\Gamma_4^{\pm} \otimes \Gamma_4^{\pm} = \Gamma_1^+ \otimes \Gamma_2^+ \otimes \Gamma_3^+$, which does not contain the irrep Γ_2^- and, consequently, provides $\mu_{n,\vec{k}} = 0$. For the Q valleys, with symmetry group C_2, the conduction bands are two-fold degenerate with imaginary (double group) irreps $\Gamma_3 \oplus \Gamma_4$ ($\Gamma_3^* = \Gamma_4$) and the operator $z \sim \Gamma_1$. Evaluating the direct product of the energy band irreps we find $\Gamma_3^* \otimes \Gamma_3 = \Gamma_1$, $\Gamma_4^* \otimes \Gamma_4 = \Gamma_1$, and $\Gamma_3 \otimes \Gamma_4 = \Gamma_1$, which contains the irrep Γ_1, and therefore we would expect to observe $\mu_{n,\vec{k}} \neq 0$. The character and multiplication tables of the relevant symmetry groups mentioned here follow the convention of Ref. [1].

For the expression $\alpha_{n,\vec{k}}$, the double matrix element provides a nonzero contribution, regardless of the presence of the horizontal mirror plane. Therefore, if the electric dipole moments of the individual energy bands are nonzero, so will be the effective dipole moment of the intersubband transition. In particular, our symmetry analysis reveals that odd layers have $\mu_{n,\vec{k}} = 0$ and so do the Γ point bands for even layers. However, for the Q valley bands for even layers we found that $\mu_{n,\vec{k}} \neq 0$. Moreover, since the effective polarizabilities are always nonzero in both even and odd layer systems, we would expect to always observe a nonzero effective polarizability for the intersubband transitions. The key point here is to quantify the magnitude of such effects, which can only be evaluated using realistic first-principles calculations.

Supplementary Note 5: DFT calculations with an out-of-plane electric field

The inclusion of electric field effects within DFT follows the implementation of the WIEN2k code [2]. In the formalism discussed in Ref. [3], the input parameter is the electrostatic potential energy, V_0, that generates the zigzag potential with $V(0) = V(c) = +\frac{V_0}{2}$ and $V(\frac{c}{2}) = -\frac{V_0}{2}$, in which c is the total size of the system in the out-of-plane direction. In our case, the size for the *N* layer system is $L_N = L_{\text{vac}} + Nd + (N-1)i$, in which $L_{\text{vac}} = 60$ Å is the vacuum region fixed for all layer numbers, $d = 3.440$ Å is the monolayer thickness and $i = 3.073$ Å is the interlayer distance. The values of d and i are taken from Ref. [4]. The WSe_2 multilayer structures are centralized at $c/4$. Conversion of the input electrostatic potential energy to electric field follows $F_z = \frac{1}{\epsilon_{\text{WSe}_2}} \frac{2V_0}{c}$, in which we incorporate the dielectric constant of WSe_2 to be consistent with the experimental definition of the electric field. We note that within our calculations the ions remain fixed and do not move under the influence of the external electric field.

The electric field has a different impact on the energy bands of the multilayered systems and, therefore, a closer look at the distinct and relevant regions is important. The experimental data is available for the conduction bands at the Q valley and the valence band at the Γ valley for all the layers studied. Moreover, signatures of the valence bands at the K valley are visible in the 3L sample. There is no experimental signature of intersubband transitions for the conduction band at the K valley for any layers and for the valence bands at the K valley for 4L, 5L, 6L, and 7L systems. We note that for the even-numbered layers 4L and 6L, the electric field calculations introduce a tiny artificial splitting in the energy levels since this breaks the intrinsic inversion symmetry. In fact, the inclusion of the electric field breaks the intrinsic symmetry for both even and odd layers. The odd-numbered layers belong to the D_{3h} point group while the even-numbered layers belong to the D_{3d} point group without an out-of-plane electric field. With the inclusion of an electric field, all systems belong to the C_{3v} point group. We note that this artificial energy splitting in even-numbered layers is rather small and does not impact the relevant physical effects, as recently shown for the natural bilayer WSe_2 [5]. Furthermore, all the observed features summarized by the effective electric dipole moments and effective polarizabilities are in excellent agreement with the expectations based on arguments of perturbation theory and group theory.

Supplementary Note 5.1 Layer decomposition of the wavefunction of CB(Q) and VB(Γ) bands

To understand the appearance of the strong linear feature in the Q bands of the 4L system, we analyze the layer decomposition of the wavefunction considering the information contained within the atomic spheres. In Supplementary Fig. 20, we show the wavefunction decomposition for each individual layer. We focus on the four lowest energy bands, labeled cQ_1 to cQ_4, in ascending energy order. Our calculations reveal that the cQ_4 and cQ_3 bands in the 4L system are strongly localized in the outer layers of the system, leading to a large electric dipole moment. For odd-layer-number systems, the degree of wavefunction localization above and below the central layer is the same, thus leading to zero electric dipole moment, consistent with our symmetry analysis.

We also evaluate the layer decomposition for the valence bands at the Γ point. The results are presented in Supplementary Fig. 21 for the wavefunction decomposition for each individual layer. We focus on the two upper-most valence bands, labeled vG_1 to vG_2, in descending energy order. Each of these bands is two-fold degenerate at the Γ point. Unlike the Q bands, the layer decomposition for even-numbered layers is nearly the same, therefore leading to very small electric dipole moments.

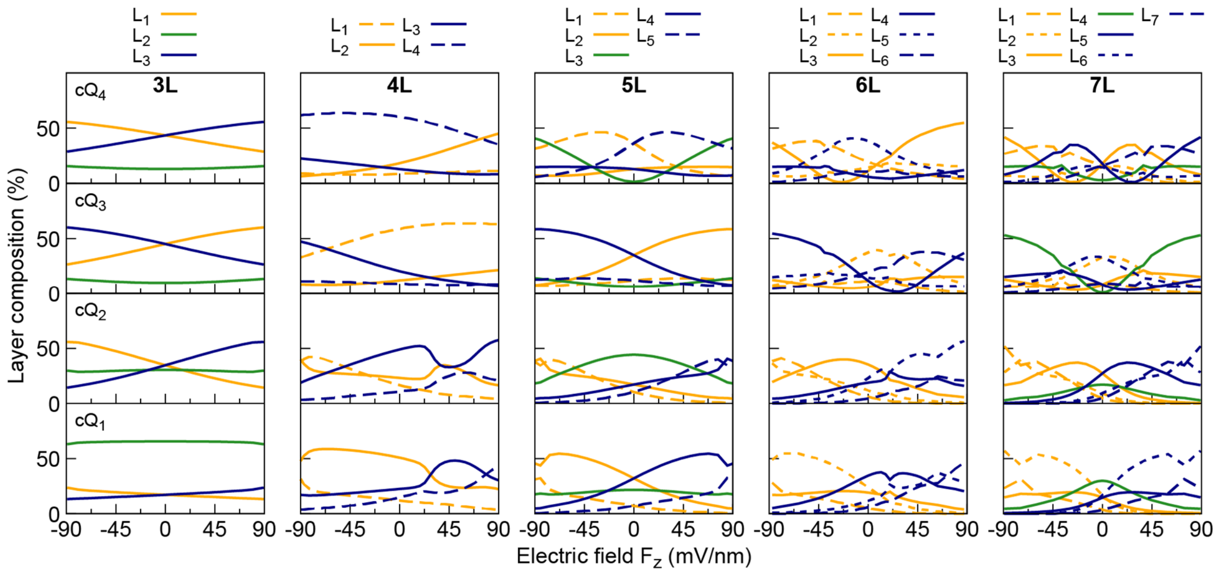

Supplementary Figure 20 | Layer decomposition of the wavefunction for conduction bands at the Q valley as a function of the external electric field.

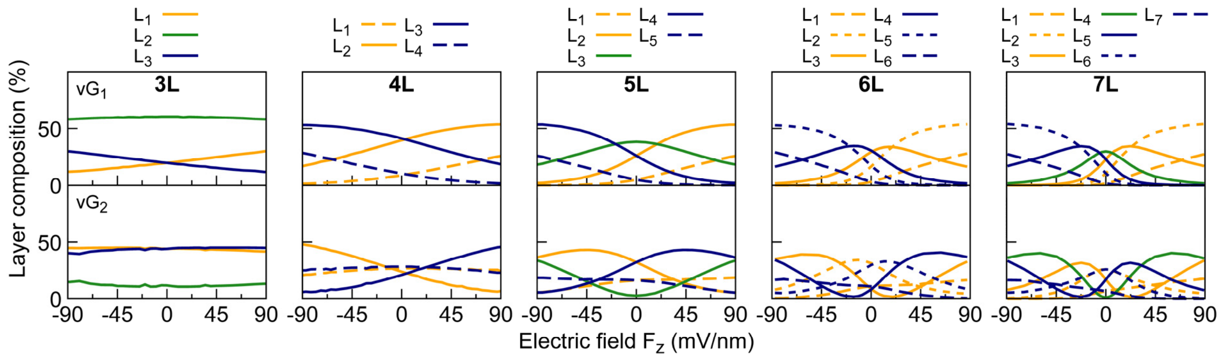

Supplementary Figure 21 | Layer decomposition of the wavefunction for valence bands at the Γ valley as a function of the external electric field.

Supplementary Note 5.2 Dipole moments and polarizabilities extracted from calculations

The electric field dependence of the lowest-energy intersubband transitions is modeled using equation (1), setting $F_{z0} = 0$ and E_0 equal to the transition energy at zero field. The values of μ and α strongly depend on the electric field range considered, indicating that the quadratic form of Eq. 1 is not fulfilled at larger fields. We summarize below the results considering different values of the maximum electric field strength ($|F_z|_{\max}$). Supplementary Fig. 22 shows the fitting for the conduction bands at the Q valley, Γ point, and K point, using $|F_z|_{\max} = 60$ meV, with the resulting fit parameters summarized in Supplementary Table 2. Supplementary Figs. 23, 24, and 25 show the individual parameter values obtained for $|\mu|$ and α using three different fit ranges $|F_z|_{\max}$. We note that our fitting procedure yields $\mu = 0$ for all the odd layer systems, in agreement with group theory expectations outlined in Section 4.3. However, we found a nonzero value of μ for the Γ bands of the 4L and 6L cases, apparently contradicting our symmetry analysis. This inconsistency arises due to the artificial symmetry-breaking of even layers when the electric field is applied (from D_{3d} to C_{3v} point groups, discussed in the beginning of Supplementary Note 5). Moreover, we note that the magnitude of μ for the Γ point bands is one to two orders of magnitude smaller than the values of μ found for the Q valley bands, and consequently these nonzero values of μ for the Γ bands can be interpreted as numerical instabilities.

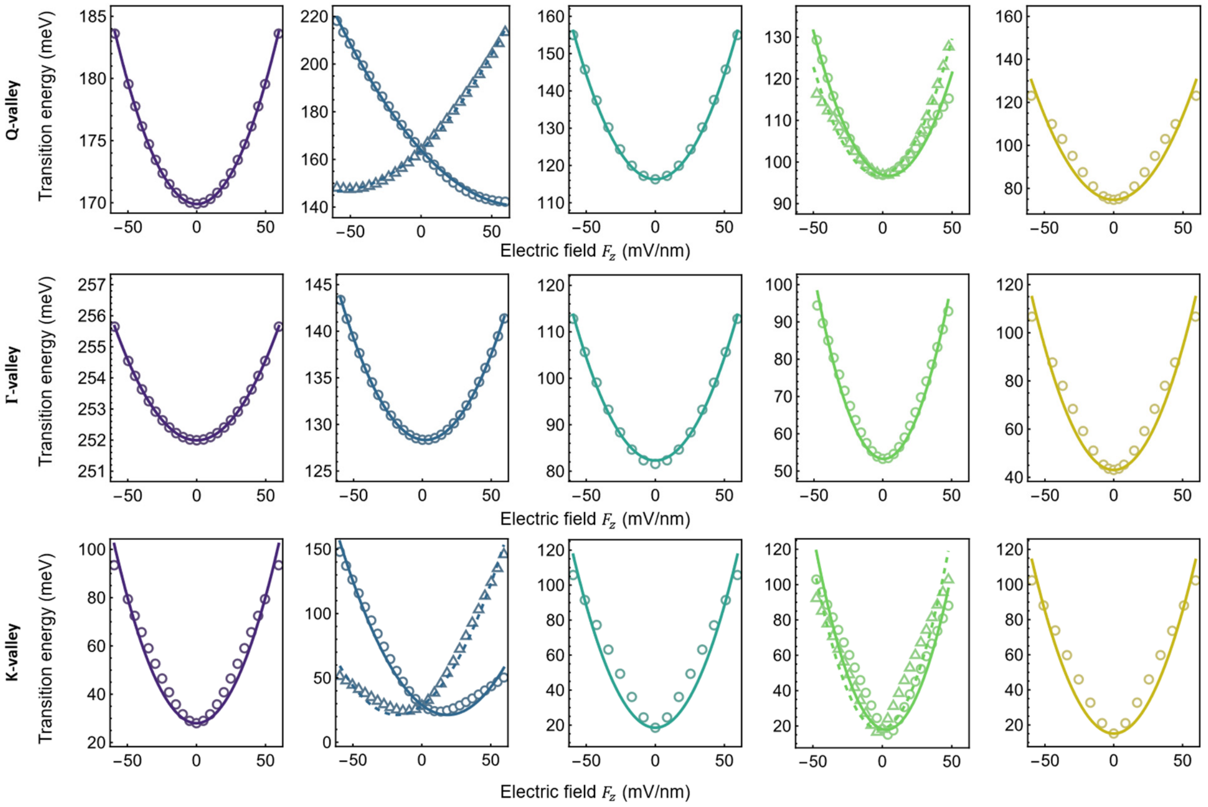

Supplementary Figure 22 | Polynomial fits of the lowest calculated intersubband transition energy as a function of the nominal out-of-plane electric field, for $|F_z|_{\max} = 60$ mV/nm. Results for the CB(Q) valley (top row), the VB(Γ) point (center row) and the VB(K) valley (bottom row). Solid (dashed) fits for 4L and 6L Q valley data correspond to the positive (negative) branches of the linear field dependence associated with the two spin-allowed transitions.

Number of layers / Valley	E_0 in meV	$\|\mu\|$ in $e \cdot nm$	α in $eV \cdot nm^2/V^2$
3L / VB(K)	28.0	0.00(3)	42(1)
3L / VB(Γ)	252	0.00000(4)	2.067(2)
3L / CB(Q)	169	0.0000(5)	7.81(2)
4L / VB(K)	30.2	0.81(1)	44.0(7)
4L / VB(Γ)	128	0.0172(5)	8.02(2)
4L / CB(Q)	164	0.614(4)	9.3(2)
5L / VB(K)	18.5	0.00(7)	56(3)
5L / VB(Γ)	81.5	0.000(6)	18.3(2)
5L / CB(Q)	116	0.000(6)	22.5(2)
6L / VB(K)	18.2	0.19(5)	81(3)
6L / VB(Γ)	53.3	0.02(1)	38.6(7)
6L / CB(Q)	96.9	0.084(7)	23.6(4)
7L / VB(K)	15.1	0.00(7)	56(3)
7L / VB(Γ)	43.0	0.00(4)	41(2)
7L / CB(Q)	69.1	0.00(4)	48(2)

Supplementary Table 2 | Parameters of the polynomial fits for the theoretical data up to $|F_z|_{\max} = 60$ meV: lowest intersubband transition energy E_0 at zero electric field, and first and second-order Stark shift parameters μ (effective dipole) and α (effective polarizability). The standard fit error for the last digit of the parameters is stated in parentheses. For 4L and 6L valleys VB(K) and CB(Q), reported values for $|\mu|$ and α are averaged over both spin-allowed branches.

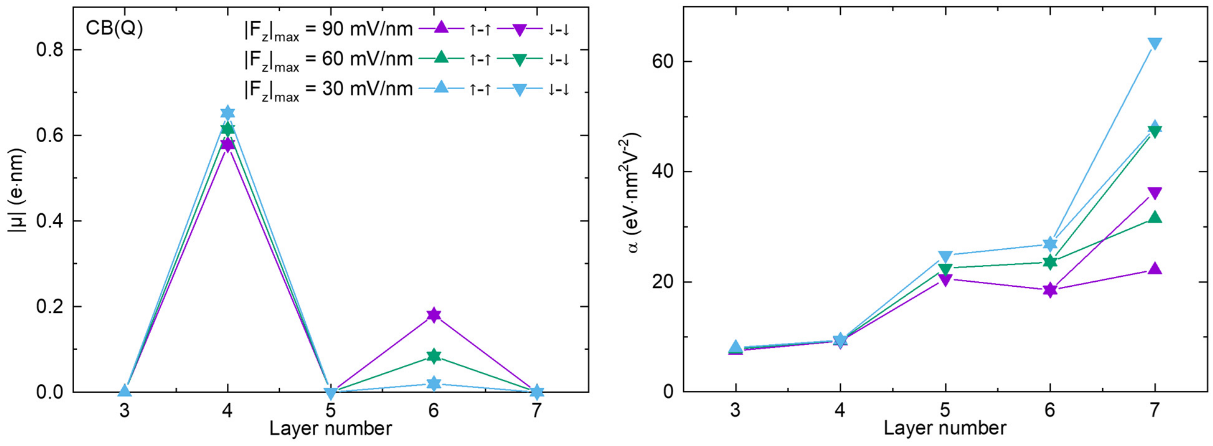

Supplementary Figure 23 | Fitting parameters $|\mu|$ and α for the lowest-energy intersubband transitions for conduction bands at the Q valley.

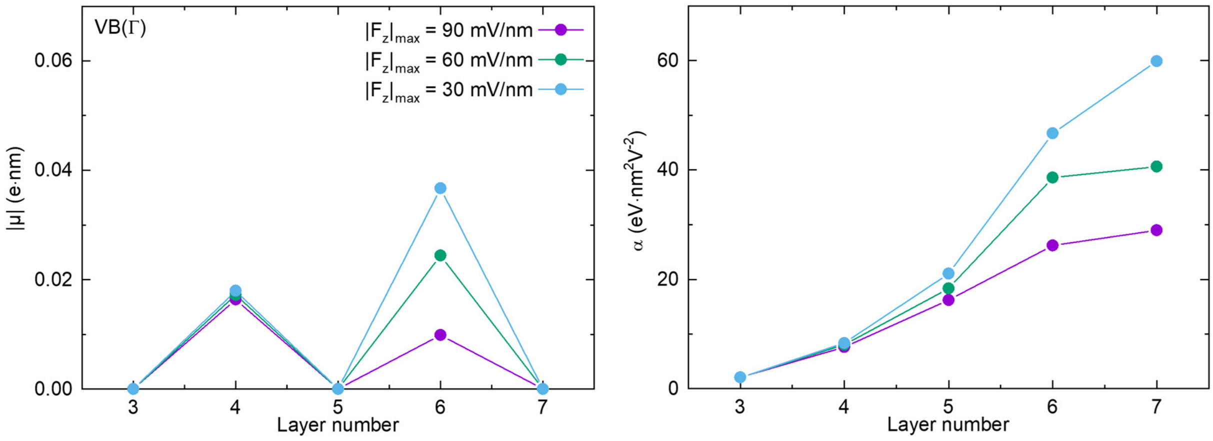

Supplementary Figure 24 | Fitting parameters $|\mu|$ and α for lowest-energy intersubband transitions for conduction bands at the Γ point.

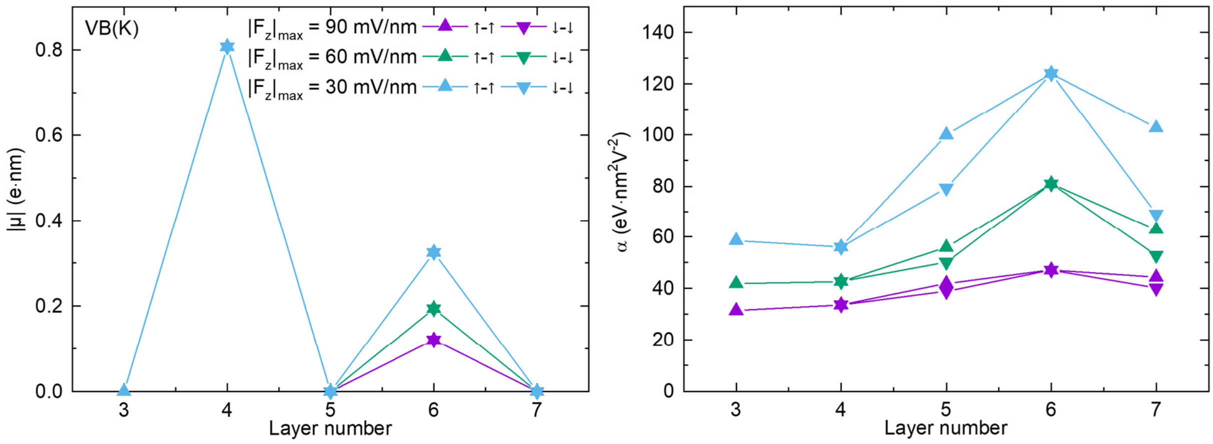

Supplementary Figure 25 | Fitting parameters $|\mu|$ and α for lowest-energy intersubband transitions for valence bands at the K point.

Supplementary Note 6: Micrographs of the twisted- and natural-multilayer WSe_2 devices

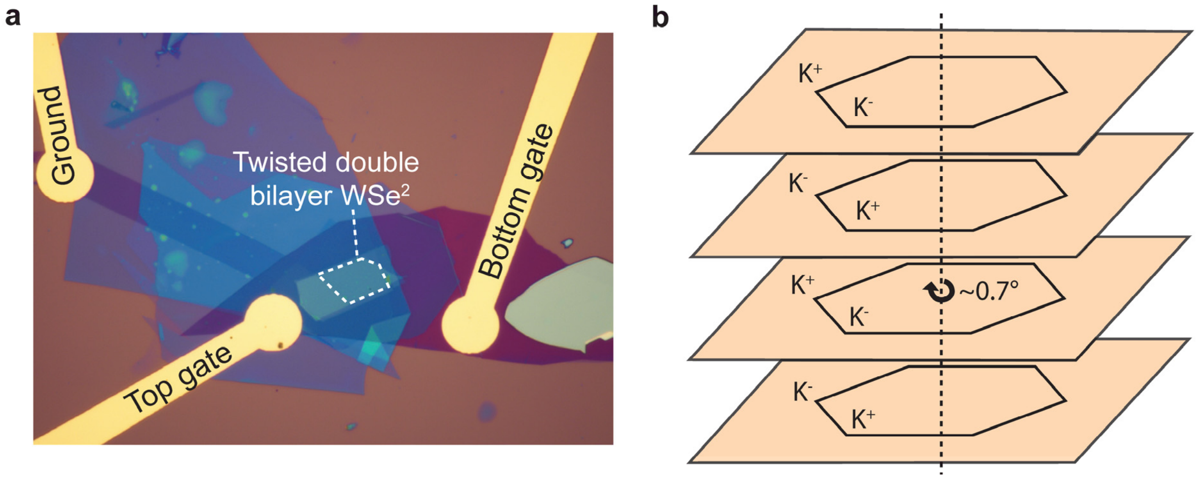

Supplementary Figure 26 | a, Micrograph of the twisted bilayer-bilayer WSe_2 device. The top and bottom hBN layer thicknesses are 17.3 nm and 11.8 nm, respectively, as measured by atomic force microscopy. **b,** Schematic depiction of the twisted bilayer-bilayer structure. The top bilayer is twisted by approximately 0.7° with respect to the bottom bilayer.

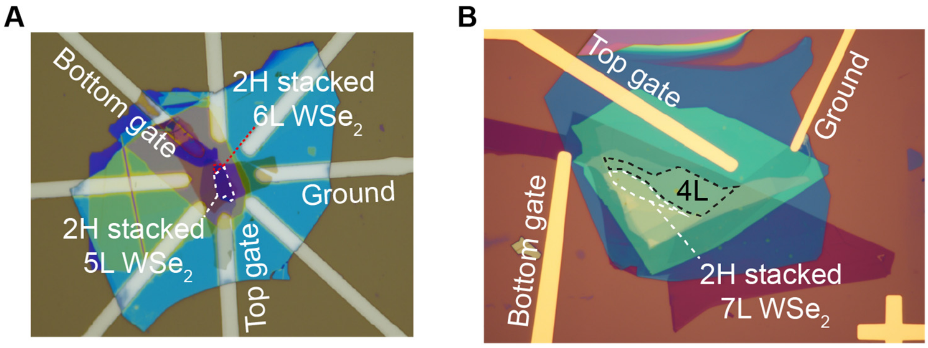

Supplementary Figure 27 | a, Micrograph of the 5L/6L WSe_2 device. Top and bottom hBN layer thicknesses are 28 nm and 29 nm, respectively, as measured by atomic force microscopy. **b,** Micrograph of the 7L WSe_2 device with a large 4L region right next to it. The top and bottom hBN layer thicknesses are 24 nm and 20.5 nm as determined by atomic force microscopy.

Supplementary References

1 Koster, G.F.; Dimmock, J.O.; Wheeler, R.G. & Statz, H. Properties of the thirty-two point groups. *MIT Press: Cambridge, MA, USA*, Volume **24** (1963).

2 Blaha, P. *et al.* WIEN2k: An APW+lo program for calculating the properties of solids. *J. Chem. Phys.* **152**, 074101 (2020).

3 Stahn, J., Pietsch, U., Blaha, P. & Schwarz, K. Electric-field-induced charge-density variations in covalently bonded binary compounds. *Phys. Rev. B* **63**, 165205 (2001).

4 Kim, H. & Choi, H.-J., Thickness dependence of work function, ionization energy, and electron affinity of Mo and W dichalcogenides from DFT and GW calculations. *Phys. Rev. B* **103**, 085404 (2021).

5 Lin, K.-Q. *et al.* Ultraviolet interlayer excitons in bilayer WSe_2. *Nat. Nanotech.* **19**, 196-201 (2024).
